# Effects of lawsone and lawsone methyl ether on cyclooxygenase activity, redox balance, and organ-specific toxicity: An integrated computational and experimental study

**DOI:** 10.1016/j.toxrep.2026.102345

**Published:** 2026-09-22

**Authors:** Muhammad Khan, Asad Ur Rahman, Phisit Khemawoot, Muhammad Ajmal Shah, Zaheer Ul-Haq, Nadeem Ahmad, Tehmina Sohail, Pharkphoom Panichayupakaranant

**Affiliations:** a Department of Pharmacology, Faculty of Pharmacy, Federal Urdu University of Arts, Sciences, and Technology, Karachi, 75300, Pakistan; b Chakri Naruebodindra Medical Institute, Faculty of Medicine Ramathibodi Hospital, Mahidol University, Samut Prakan, 10540, Thailand; c Department of Pharmacy, Hazara University, Mansehra, 21300, Pakistan; d Dr. Panjwani Center for Molecular Medicine and Drug Research, International Center for Chemical and Biological Sciences, University of Karachi, Karachi, 75270, Pakistan; e Pharmaceutical Research Center, Pakistan Council of Scientific & Industrial Research (PCSIR), Laboratories Complex, Karachi, 75280, Pakistan; f Department of Pharmacognosy and Pharmaceutical Botany, Faculty of Pharmaceutical Sciences, Prince of Songkla University, Hat-Yai, 90112, Thailand; g Phytomedicine and Pharmaceutical Biotechnology Excellence Center, Faculty of Pharmaceutical Sciences, Prince of Songkla University, Hat-Yai, 90112, Thailand

**Keywords:** Acute toxicity, COX-inhibition, Lawsone, Lawsone methyl ether, LD_50_, Redox-modulation, Subacute toxicity

## Abstract

Redox imbalance contributes significantly to the initiation and progression of inflammatory responses. Although conventional anti-inflammatory drugs are widely used, their prolonged administration is associated with a number of adverse effects. Lawsone methyl ether (LME) and lawsone are plant-derived, redox-active naphthoquinones, exhibiting diverse pharmacological activities; however, their cyclooxygenase (COX)-inhibitory effects, redox-modulatory properties, and oral toxicity profiles remain insufficiently characterized. Therefore, this study investigated the COX-inhibitory activity, redox-modulatory effects, and oral toxicity profiles of LME and lawsone using integrated computational, enzyme-inhibitory, and *in vivo* approaches. Molecular docking analysis revealed favorable binding scores/interactions of LME against COX-1 (−5.9 kcal/mol), lipoxygenase (LOX; −6.6 kcal/mol), NAD(P)H oxidase (−5.6 kcal/mol), and myeloperoxidase (MPO; −4.7 kcal/mol), surpassing lawsone and reference inhibitors (aspirin, and zileuton). These results were further validated by molecular dynamics (MD) simulations. Additionally, LME exhibited higher *in vitro* inhibition of COX-1 (IC_50_: 8.1 µM) and COX-2 (IC_50_: 4.2 µM) than lawsone. Furthermore, acute (50/300 mg/kg) and subacute (50 mg/kg) oral administration of LME in Wistar rats significantly ameliorated glutathione (GSH), superoxide dismutase (SOD), catalase (CAT), and thiobarbituric acid-reactive substance (TBARS) levels, more effectively than lawsone. Following Karber’s method, oral LD_50_ values calculated for LME and lawsone were 542 and 175 mg/kg, respectively. However, in a 28-day repeated oral toxicity study, a no-observed-adverse-effect level (NOAEL) of at least 50 mg/kg was established for both the compounds, with no toxicity observed across various rat parameters. Overall, LME was better tolerated than lawsone, exhibiting COX inhibitory and redox-modulatory effects, but its therapeutic significance requires further validation.

## Highlights

• Safety and redox-modulatory assessment of lawsone and lawsone methyl ether (LME).
• LME and lawsone exhibited acute oral LD_50_ of 542 and 175 mg/kg, respectively.
• 15, 30, and 50 mg/kg of oral LME or lawsone were found safe in rats for 28 days.
• LME inhibited COX-1/2, showing synergistic effects with indomethacin and celecoxib.
• LME reduced redox-imbalance by alleviating TBARS, GSH, CAT, and SOD levels in rats.

## Graphical Abstract

**Figure:**
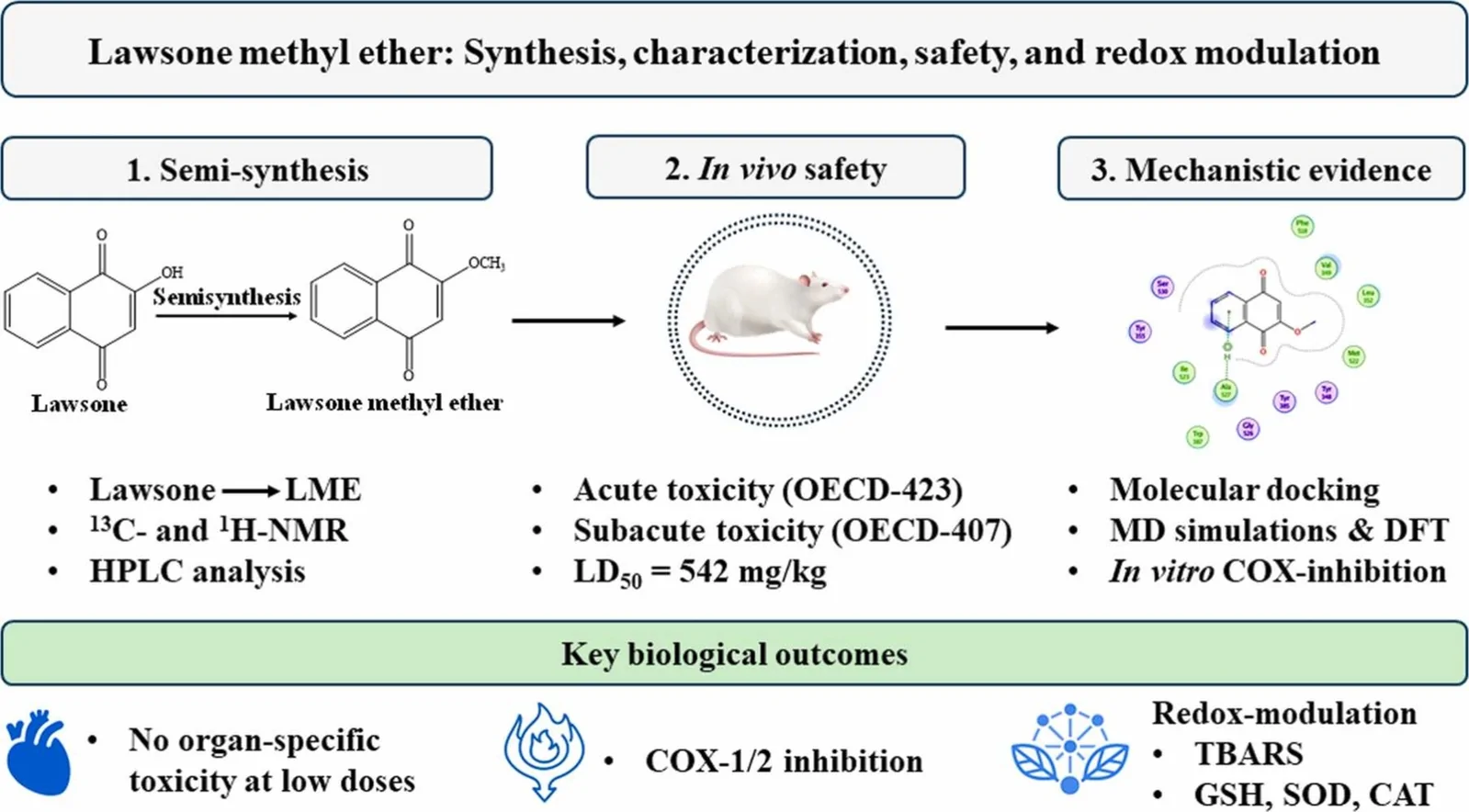

## 1 Introduction

Oxidation–reduction (redox) reactions are central to life, playing a pivotal role in cellular physiology. Reactive oxygen and nitrogen species (RONS) are important mediators of redox regulation [1]. Normally, redox homeostasis is tightly maintained; however, when pro-oxidant species breach the antioxidant defenses, this balance is disturbed, mounting excessive oxidative stress. Such an imbalance can deteriorate a number of cellular macromolecules, specifically deoxyribonucleic acid (DNA), subsequently leading to genetic or epigenetic alterations. Therefore, maintaining a fine-tuned and steady redox state is essential for preserving cellular viability [2]. To counteract RONS, a biological system relies on various endogenous antioxidants, including SOD, CAT, GSH, GPx (glutathione peroxidase), and GSR (glutathione reductase) [3]. Notably, redox imbalance is the central feature of numerous chronic diseases, playing a key role in the onset and progression of redox-inflammatory conditions [4].

Inflammation is a complex biological response to detrimental stimuli, including injury, infection, or irritants, aimed at restoring tissue repair and homeostasis [5]. Oxidative stress and inflammation are two interrelated phenomena; however, their persistent infliction can induce lasting damage to healthy tissues, leading to cardiovascular, respiratory, neurodegenerative, metabolic, and hepatorenal diseases [6]. In this context, a number of anti-inflammatory agents, such as steroids, COX-inhibitors, tumor necrosis factor (TNF) inhibitors [7], interleukin (IL) blockers, and leukotriene modifiers [8] are conventionally available. Nonetheless, their long-term consumption or overdosing has been associated with gastrointestinal, cardiovascular, reproductive, neuronal, or hepatorenal toxicities [[9], [10], [11]].

The aforementioned limitations of conventional anti-inflammatory medications have led to the discovery of novel plant-based naphthoquinones (NQs) with notable COX-inhibitory and redox-modulatory potentials. Among them, juglone, plumbagin, shikonin, diospyrin, and lapachol are the prominent examples [12]. Similarly, LME (2-methoxy, 1,4-NQ) and lawsone (2-hydroxy, 1,4-NQ), as shown in Fig. S1 **(Supplementary file)**, are the plant-based NQs, primarily found in the leaves of *Impatiens balsamina* L. [13]. Both of them possess *in vitro* antioxidant properties [14,15]. Additionally, LME has been reported to exhibit antiallergic [16], antiplatelet aggregation [17], and neural repair [18] activities. Meanwhile, lawsone has been found to showcase immunomodulatory [15], antisickling [19], and hepatoprotective [20] potentials. Furthermore, our previous research uncovered hypoglycemic, hypolipidemic, and hepatorenal protective effects of LME as well as lawsone in streptozotocin-induced diabetic rats [13,21]. Nowadays, lawsone is commercially available and its methylation can synthesize LME with high yield [22]. This semisynthetic process offers additional advantages over traditional plant extraction or isolation methods in terms of affordability, scalability, purity, and convenience. All these factors are crucial for pharmaceutical development, paving the way for the formulation of lead compounds with improved therapeutic accessibility.

Despite a number of biological activities, commercial availability, and scalable synthesis, neither LME nor lawsone has been investigated for its COX-inhibitory, redox-modulatory, and oral toxicity attributes. To the best of our knowledge, this study is the first-ever report elucidating the impact of LME and lawsone on COX-1, COX-2, GSH, SOD, CAT, and TBARS using integrated computational, enzyme-based, and *in vivo* models. Furthermore, this is the first study that highlights acute and subacute toxicities of LME and lawsone in rodent models, following the Organization for Economic Co-operation and Development (OECD) 423 and 407 guidelines, respectively.

## 2 Materials and methods

### 2.1 Drugs and chemicals

Lawsone, Triton X–100 (TX–100), Elman’s reagent or 5,5′-dithiobis-(2-nitrobenzoic acid) (DTNB), Griess reagent, dibasic/monobasic sodium phosphate, methanol, ethanol, butylated hydroxytoluene (BHT), trichloroacetic acid (TCA), thiobarbituric acid (TBA), hydrochloric acid (HCl), hydrogen peroxide (H_2_O_2_), nitroblue tetrazolium (NBT), phosphate-buffered saline (PBS), dimethyl sulfoxide (DMSO), ethyl acetate, hexane, chloroform, deuterochloroform (CDCl_3_), epinephrine, sodium carbonate (Na_2_CO_3_), phosphoric acid, copper sulfate, and all the other chemicals/reagents were purchased from Sigma-Aldrich Chemie GmbH (Steinheim, Germany).

### 2.2 Semisynthesis, NMR characterization, and HPLC quantification of LME

Semisynthesis of LME was performed in-house using lawsone in a single batch as previously described [13]. The semisynthetic LME was subsequently characterized by nuclear magnetic resonance (NMR) spectroscopy, thin-layer chromatography (TLC), and high-performance liquid chromatography (HPLC). ^1^H and ^13^C NMR spectroscopy was performed following a previous protocol [23]. However, TLC was conducted using three different solvent systems of varying polarities: hexane/chloroform (7:3), chloroform/methanol (5:5), and hexane/ethyl acetate (6:4). Following an already established method [24], HPLC quantification was performed under the conditions of a binary HPLC pump (Waters 1525) equipped with a photodiode array detector (Waters 2998, USA), an auto-sampler (Waters 2707), and an ACE 5 C18-PFP column (150 × 4.6 mm, 5 µm). The injection of sample volume was 20 µL, and the column was eluted isocratically with a solution of acetonitrile and 0.2% formic acid at a ratio of 70:30 v/v, and a flow rate of 1 mL/min.

### 2.3 *In silico* toxicological and anti-inflammatory prediction

#### 2.3.1 *In silico* acute toxicity prediction via online web-servers

*In silico* acute systemic and topical toxicities of LME and lawsone were predicted using three independent online web-servers, namely, STopTox, accessed via https://stoptox.mml.unc.edu/ [25], ADMETlab 3.0, accessible at https://admetlab3.scbdd.com/ [26], and the EPA’s Toxicity Estimation Software Tool (TEST) 5.1.2 available at https://www.epa.gov/comptox-tools/toxicity-estimation-software-tool-test [27].

#### 2.3.2 Molecular docking analysis against redox-inflammatory targets

The crystallographic structures of COX-I (PDB ID: 3KK6; Resolution 2.75 Å), LOX (PDB ID: 1N8Q; Resolution 2.10 Å), nicotinamide adenine dinucleotide (phosphate) oxidase (NAD(P)H oxidase, PDB ID: 2CDU; Resolution 1.80 Å), and MPO (PDB ID: 1DNU; Resolution 1.85 Å) were downloaded from the RCSB Protein Data Bank (https://www.rcsb.org/). The structures of target proteins were optimized using Molecular Operating Environment (MOE 2019; Chemical Computing Group, Montreal, QC, Canada). In this step, the removal of crystallographic water molecules and non-essential heteroatoms was followed by energy minimization and assignment of partial charges. Importantly, the reference ligands co-crystallized with the protein targets, such as celecoxib (3KK6), protocatechuic acid (1N8Q), FAD: Flavin adenine dinucleotide (2CDU), and thiocyanate (1DNU), were retained to facilitate the Induced Fit docking protocol. However, all the other cofactors and metals (including heme) were removed from the crystal structures of the target proteins. On the contrary, the two-dimensional (2D) structures of LME, lawsone, and the reference inhibitors (aspirin, zileuton, and resveratrol) were retrieved from the PubChem database (http://pubchem.ncbi.nlm.nih.gov/). After their conversion to 3D structures via ChemOffice^+^ 22.0 (PerkinElmer), they were energy-minimized (MMFF94 force field) using MOE software.

The docking protocol was validated by redocking the co-crystallized ligand into the active site of COX-1 (PDB ID: 3KK6). The overlaid crystallographic and redocked ligand postures are shown in Fig. S2 (**Supplementary file**). Wherein the redocked binding pose exhibited a root mean square deviation (RMSD) of 0.6 Å, when compared to the crystallographic conformation, validating the authenticity of the docking protocol. Notably, the docking calculations were performed using the binding site defined by the coordinates of the co-crystallized ligand. Finally, MOE was run to perform molecular docking analysis, as previously described [28]. The resulting 3D ligand–protein interactions were visualized via Protein-Ligand Interaction Profiler [29].

#### 2.3.3 Molecular dynamics (MD) simulations

MD simulations were performed using GROMACS to predict the structural stability, residue-level flexibility, and overall compactness of protein–ligand complexes [30]. Based on docking results, four complexes, LME–COX, LME–LOX, LME–NAD(P)H oxidase, and LME–MPO, alongside their respective apo proteins, were selected for the simulation. Protein topologies were generated via the AMBER99SB force field [31]. Conversely, the ligand topologies were prepared using ACPYPE [32]. Additionally, each system was solvated using a cubic periodic boundary box with the simple point charge (SPC) water model; however, neutralization was performed by adding Na⁺ and Cl⁻ counter ions to simulate a physiological condition [33]. Energy minimization was performed using the steepest-descent algorithm for 5000 steps to eliminate steric hindrances and unfavorable contacts. Afterward, the systems were equilibrated at 300 K and 1 bar under NVT (constant volume and temperature) and NPT (constant pressure and temperature) ensembles, employing a thermostat relaxation time of 1000 ps [34,35]. Long-range electrostatic interactions were computed using the Particle Mesh Ewald (PME) method, covalent bonds involving hydrogen atoms were restricted with the help of the LINCS algorithm, and all the simulations employed a 2 fs integration time step. Finally, 100 ns production MD simulations were conducted for all systems, and the resulting trajectories were analyzed to assess structural stability, residue-level flexibility, and compactness using RMSD, root-mean-square fluctuation (RMSF), and radius of gyration (Rg), respectively.

#### 2.3.4 Density functional theory (DFT) calculations

DFT calculations were performed to predict the electronic reactivity and structural robustness of LME and lawsone. In this study, the electronic structures of both the ligands were described via electron density rather than the many-electron Schrödinger equation [36]. Herein, the ORCA quantum chemistry software package was used to calculate the highest occupied molecular orbital (HOMO), lowest unoccupied molecular orbital (LUMO), and their respective energy gaps. Before performing these calculations, the ligand geometries were optimized and subsequently energy-minimized via Avogadro, in order to generate the corresponding input files for DFT calculations [37]. All ORCA calculations were performed using PowerShell on Microsoft Windows 11 (64-bit). The resulting output files were analyzed, and the HOMO and LUMO orbital distributions were visualized using IboView, following the previously reported protocol [38].

### 2.4 *In vitro* cyclooxygenase (COX-1 and COX-2) inhibition assay

The *in vitro* COX inhibitory assay was performed as previously described [39], with a few modifications, using ovine COX-1 and human recombinant COX-2 Inhibitor Screening Assay Kit obtained from Cayman Chemical (Ann Arbor, MI, USA, Cat. No. 560131). For each experiment, the samples were freshly prepared in DMSO, ensuring that the final DMSO concentration did not exceed 7%. Before each use, dosing homogeneity of the samples was ensured by vortexing or sonication at a temperature not exceeding 37 **°**C. Briefly, the reaction mixture consisted of enzyme (10 µL), heme (10 µL), and the sample solutions (20 µL). It was subsequently mixed with 160 µL of the assay buffer (pH 8.0) and left to incubate for 10 min at 37 °C. The reaction was initiated by adding 10 µL of arachidonic acid, which was stopped after 2 min by adding stannous chloride (30 µL). The final product, prostaglandin-F_2_α (PGF_2_α) produced in each reaction was quantified via an enzyme-linked immunosorbent assay (ELISA) kit. After incubation of samples in a 96-well plate for 18 h at room temperature, the wells were washed and developed using 200 µL of Ellman's Reagent. An enzyme solution deactivated by boiling (heat-inactivated enzyme) was used as a blank. To perform the control experiment, the sample contained an equivalent concentration of DMSO dissolved in deionized water. All the assays were performed in triplicate (*n* = 3), wherein indomethacin and celecoxib were used as positive controls for COX-1 and COX-2, respectively. The absorbance was read at 405 nm using Varioskan™ LUX Multimode Microplate Reader (Thermo Scientific, MA, USA). The resultant enzyme inhibition (%) was calculated by the following equation:

$$\text{Enzyme inhibition}\;\left(\%\right)=\left[\;\frac{\left(Ac-Ab\right)-(As-Ab)}{\left(Ac-Ab\right)}\right]\;\times 100$$

Where, A*c* is the absorbance of the positive control (indomethacin/celecoxib), A*b* is the absorbance of the blank, and A*s* is the absorbance of the sample compound (LME/lawsone). The IC_50_ values were calculated using GraphPad Prism (Version 10, San Diego, CA, USA) from the dose–response curves of eight graded concentrations (0.19–25 µM) for each tested compound.

### 2.5 Determination of additive, synergistic, or antagonistic interactions via isobolographic analysis

Inhibitory interactions of LME or lawsone combined with indomethacin and celecoxib against COX-1 and COX-2 were performed following the aforementioned protocol [39]. The combined inhibitory activity was investigated using the fraction of IC_50_ values. Herein, three IC_50_ fractions (½IC_50_, ¼IC_50_, and ⅛IC_50_) of LME or lawsone were combined with those of indomethacin or celecoxib. Consequently, a total of 9 combinations (C1–C9) were formulated for each naphthoquinone (LME or lawsone) and each of indomethacin or celecoxib. The percentage inhibition of COX-1 and COX-2 activities for each combination was calculated as described earlier.

The interactions of LME or lawsone with indomethacin and celecoxib were interpreted via isobolographic analysis using 50% enzyme inhibition as a predefined effect level [40,41]. Briefly, the isobolograms were generated by plotting the IC_50_ of indomethacin or celecoxib on the y-axis and the corresponding IC_50_ of LME or lawsone on the x-axis. Both these IC_50_ values were connected by a theoretical straight line called additivity line or 50% isobole. The experimentally tested combinations were subsequently plotted as coordinate pairs across that isobologram. Combination points located below the line, exhibiting ≥ 50% enzyme inhibition, were termed synergistic; however, the points located on the line were interpreted as additive. The points located above the line were considered antagonistic. Notably, the combinations that failed to induce at least 50% enzyme inhibition were deemed inconclusive and not assigned a definitive classification.

### 2.6 *In vitro* hemocompatibility assay

The *in vitro* hemocompatibility of LME and lawsone was assessed via a hemolysis assay [42], after being approved by the Institutional Ethical Committee (IEC) of the Pakistan Council of Scientific & Industrial Research (PCSIR), Laboratories Complex, Karachi, Pakistan, with the protocol number: PCSIR–KLC/IEC/2022/01. Additionally, a written informed consent was obtained from all the blood donors. Blood samples from five healthy donors were analyzed individually rather than pooled. Briefly, the samples were centrifuged (2000 × g, 10 min, and 4 °C) to obtain red blood cell (RBC) pellets, and subsequently suspended in PBS. Test compounds were separately incubated with the RBC suspension and centrifuged (1200 × g, 3 min) to obtain a supernatant. The absorbance of hemoglobin released into the supernatant was measured at 578 nm using Varioskan™ LUX Multimode Microplate Reader (Thermo Scientific, MA, USA). Each experiment was performed in triplicate (three technical replicates). Concentrations of (5, 10, and 20 µM) LME/lawsone were selected as a two-fold dilution series within the low-micromolar range, based on the concentrations previously reported to exert biological activities *in vitro* [14,43,44]. However, Triton X-100 (1% v/v) was used as a reference, defining maximal (100%) hemolysis, whereas, PBS alone served as the negative control (0% hemolysis). To optimize compound-associated absorbance, at first, the compound blanks (corresponding compound concentration in PBS without RBCs) were measured at 578 nm and then their absorbance values were subtracted from the respective test-sample readings. The percent hemolytic activity was calculated by the following formula:

$$\mathrm{Hemolysis}\left(\%\right)=\;\frac{A_{\mathrm{sample}\;-\;A_{\mathrm{negative}}\;}}{A_{\mathrm{positive}\;-\;A_{\mathrm{negative}}\;}}\;\times 100$$

Where, A_sample_, A_negative_, and A_positive_ are the absorbances of sample, PBS, and Triton X-100, respectively.

### 2.7 *In vivo* oral toxicity and redox-modulatory assay

#### 2.7.1 Experimental animals and housing conditions

A total of 88 Wistar rats (53 females and 35 males), approximately 7–8 weeks old and weighing 200 ± 20 g, were obtained from the PCSIR, Laboratories Complex, Karachi, Pakistan. The distribution of animals across the acute and subacute toxicity experiments is summarized in Table S1**.** The animals were kept within standard polypropylene cages with corn cob bedding under controlled laboratory conditions (24 ± 2 °C, 55 ± 10% humidity, 12 h/12 h light/dark cycle) with *ad libitum* standard diet and water. All the experimental procedures were initiated following a one-week acclimatization period. At the conclusion of each study, the animals were euthanized by gradual-fill carbon dioxide (CO_2_) inhalation (20–30% chamber volume/min), in accordance with American Veterinary Medical Association (AVMA) guidelines.

#### 2.7.2 Ethics statement

This study was conducted in accordance with the Guide for the Care and Use of Laboratory Animals specified by the Institute of Laboratory Animal Resources (NIH publication No. 85–23). The acute and subacute oral toxicity protocols followed OECD Test Guideline No. 423 and 407, respectively. All the animal procedures were approved by the IEC of the PCSIR Laboratories Complex, Karachi, Pakistan, with the protocol number: PCSIR–KLC/IEC/2022/02 and this manuscript was prepared in accordance with ARRIVE 2.0 (Animal Research: Reporting of In Vivo Experiments) guidelines.

#### 2.7.3 Acute toxicity test–acute toxic class method

Following OECD Test Guideline No. 423, the acute toxicity of LME/lawsone was performed using female rats. The test compounds were always freshly prepared for oral administration as a micro-emulsion (propylene glycol, Tween 80, and water) via gavage (≤ 1 mL/100 g), to minimize the concern of formulation stability. Before each use, homogeneity of the dosing formulation was ensured by vortexing or a brief sonication at a temperature not exceeding 37 **°**C. The animals were randomly assigned to six groups (*n* = 3 per group): Group 1 (control) received the vehicle (cosolvent) only; Groups 2–4 received 50, 300, and 2000 mg/kg of LME, respectively; and Groups 5 and 6 received 50 and 300 mg/kg of lawsone, respectively. Dosing was initiated at 50 mg/kg and escalated to 300 mg/kg or reduced to 5 mg/kg, depending on the number of mortalities observed following administration of the initial dose. The mortality data obtained at each dose level were used to calculate the median lethal dose (LD₅₀) using Karber's method. Moreover, the hazard levels of LME and lawsone were determined based on their LD_50_ values following the Globally Harmonized System of Classification and Labelling of Chemicals (GHS).

#### 2.7.4 Subacute toxicity test

A 28-day repeated dose oral toxicity study was performed using adult male and female Wistar rats (*n* = 70) following OECD 407 Guideline [21,45]. The animals were randomly allocated to seven groups (*n* = 5 rats/sex/group): a vehicle control (cosolvent only), three LME-treated groups, and three lawsone-treated groups, each set receiving 15, 30, or 50 mg/kg/day of the respective compound. Both the compounds were repeatedly administered for 28 days via oral gavage as described earlier. Mortality, clinical observations, body weight, food/water intake, and relative organ weights were recorded as mentioned in our previous work [46]. Additionally, fasting blood glucose (FBG) levels were measured at baseline and weekly thereafter.

#### 2.7.5 Hematological and biochemical analysis

Immediately after euthanasia, blood was collected via cardiac puncture for biochemical and hematological assessment. The serum obtained from non-heparinized blood was subsequently analyzed via commercial diagnostic kits (Martin Dow, Innoline®, France), using a Stat Fax 3300 analyzer, to determine the levels of alanine transaminase (ALT), aspartate transaminase (AST), alkaline phosphatase (ALP), creatinine (Cr), uric acid, blood urea nitrogen (BUN), total bilirubin (TB), high-density lipoproteins (HDL), low-density lipoproteins (LDL), total cholesterol (TC), and triglycerides (TGs). However, hematological investigation was conducted on heparinized whole blood using an automated hematology analyzer (Genrui Biotech KT-6200, China), measuring various erythrocyte indices including, RBC count, hemoglobin, packed cell volume (PCV), mean corpuscular volume (MCV), mean corpuscular hemoglobin (MCH), mean corpuscular hemoglobin concentration (MCHC), and white blood cell (WBC) differential counts (total WBCs, lymphocytes, monocytes, eosinophils, basophils).

#### 2.7.6 Histopathological analysis

Histopathological sections of the major visceral organs (heart, liver, lungs, kidneys, and spleen) from male and female rats were prepared and analyzed as mentioned previously [47]. Histopathological analysis was performed using a two-stage analytical approach to minimize evaluator bias. Initially, an unblinded screening was conducted to identify potential treatment-related histopathological changes. This was followed by a blinded, semi-quantitative assessment to confirm and grade the severity of the identified lesions. The grading approach was adapted from a previously established method [48], wherein histopathological changes were categorized using a four-point severity scale (0 = unchanged, 1 = mild, 2 = moderate, 3 = severe). This grading system was consistently applied to assess the severity of histopathological changes across various organs and treatment groups.

#### 2.7.7 Analysis of the oxidative stress markers

Whole blood from euthanized rats was immediately collected via a terminal cardiac puncture into dipotassium ethylenediaminetetraacetate (K_2_EDTA)-coated vacuum tubes. The blood was centrifuged at 2500 × g for 15 min at 4 °C to obtain plasma for subsequent storage at –80 °C until further analysis [49]. The heart, liver, and kidneys were promptly excised after euthanasia, rinsed with ice-cold saline (0.9% NaCl), and homogenized in a 0.1 M phosphate buffer (pH 7.4) on ice. The homogenates were centrifuged at 4000 × g for 25 min at 4 °C, the resulting supernatants were aliquoted, and stored at –80 °C for subsequent analysis [50].

The levels of TBARS, GSH, SOD, and CAT were quantified in the plasma, heart, liver, and kidneys using previously established assay protocols. TBARS levels were determined via a colorimetric assay, as previously described [51]. Similarly, GSH levels were measured by reacting DTNB with sulfhydryl groups to form a yellow chromophore, which was then quantified at 412 nm [52]. Likewise, the SOD activity was assayed by following an already established protocol [53], wherein the enzyme’s capacity to inhibit NBT reduction by superoxide radicals was measured at 560 nm. Finally, the CAT activity was evaluated by monitoring the rate of decomposition of H_2_O_2_ at 240 nm [54]. All the spectrophotometric measurements were performed using a dual-beam spectrophotometer (Cary 300, Agilent Technologies, Santa Clara, CA, USA).

### 2.8 Statistical analysis

The results from *in vitro* studies were expressed as mean ± standard deviation (SD), while those from *in vivo* studies were presented as mean ± standard error of the mean (SEM). GraphPad Prism software (Version 10, San Diego, CA, USA) was utilized for all the statistical analyses. The endpoints evaluated at a single time point (determination of biochemical or oxidative stress markers) were statistically analyzed using one- or two-way ANOVA, depending on the experimental design. In contrast, the experiments involving repeated measurements over time (FBG determinations) were analyzed using a two-way (Treatment × Sex) or three-way (Treatment × Sex × Week) repeated-measures ANOVA, as appropriate. In all cases, ANOVA (one-way, two-way, or three-way) was followed by Tukey's post hoc test for multiple comparisons*.* Owing to a fixed and limited sample size (*n* = 3 rats/group) prescribed by OECD 423 Guideline, the mortality data (number of deaths per group) and histopathological severity scores were presented descriptively, rather than subject to a formal statistical comparison. In all cases, a *p*-value of less than 0.05 was defined as statistically significant.

## 3 Results and discussion

### 3.1 Semisynthesis, NMR, TLC, and HPLC characterization of LME

LME was semisynthesized from lawsone in a single batch, producing yellow, needle-shaped crystals with a yield exceeding 70%. Semisynthetic LME and commercial lawsone were stored in powdered form at room temperature in airtight amber glass vials, protected from light and moisture, within a well-ventilated storage area [55]. All the experiments reported in the current study were performed using LME or lawsone from the same batch in order to avoid batch-to-batch variability.

The structure of semisynthetic LME was confirmed by ^1^H and ^13^C NMR spectroscopy (Fig. 1). The ^13^C NMR spectrum (126 MHz, CDCl_3_) displayed eleven distinct carbon resonances, including δ 184.8 (C-4), 180.1 (C-1), 160.4 (C-2), 134.3 (C-6), 134.2 (C-7), 133.3 (C-10), 132.0 (C-9), 131.0 (C-8), 126.8 (C-5), 126.2 (C-3), and 56.4 (C-11). The remaining signals observed in the spectrum corresponded to the residual solvent (CDCl_3_) and tetramethylsilane (TMS), the internal reference standard, confirming the absence of impurities.

**Fig. 1:**
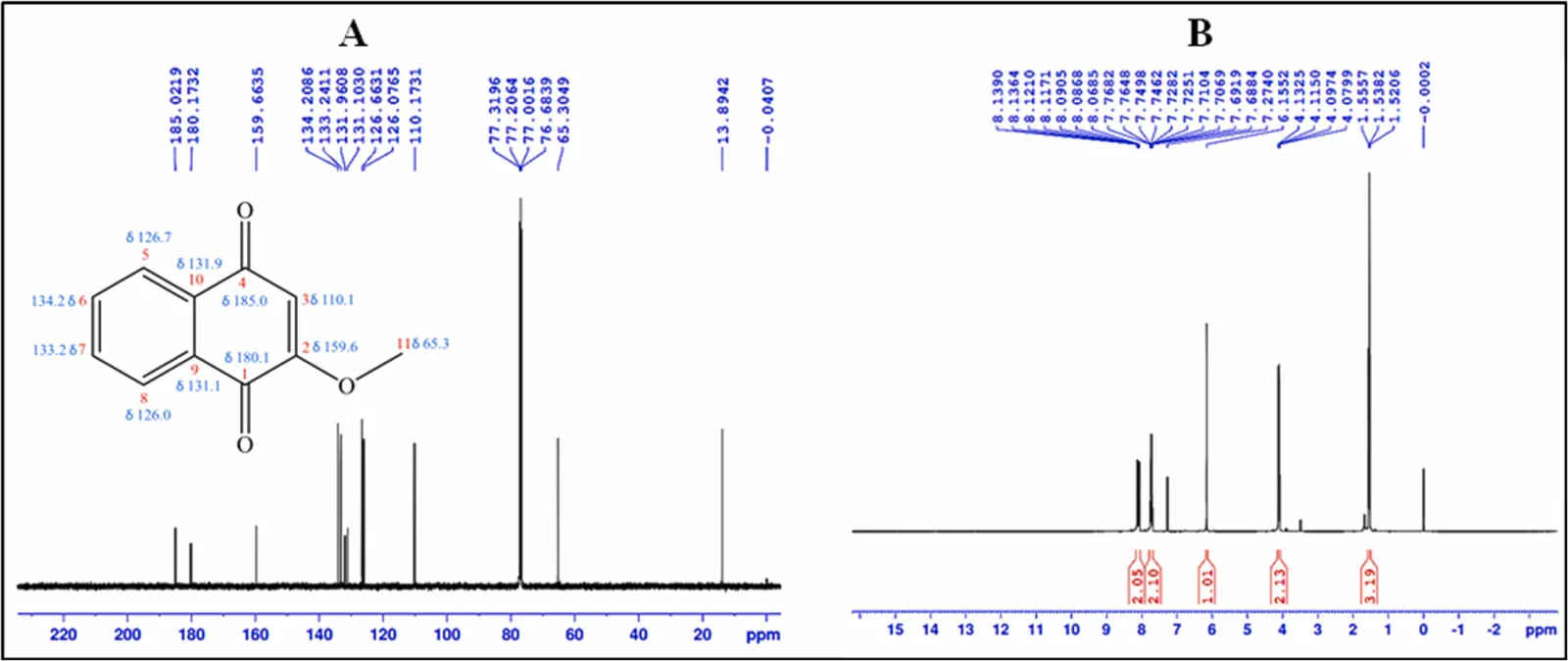
Representative (**A**) ^13^C NMR and (**B**) ^1^H NMR spectra of semisynthetic LME.

The preliminary purity assessment was performed by TLC, wherein the semisynthetic LME migrated as a single spot with an Rf (retention factor) value identical to that of the standard LME across three solvent systems of varying polarities (Fig. S3 **in Supplementary file**). Additionally, the quantification of LME was performed by HPLC, which revealed a purity of 98.0 ± 2.0% w/w based on the LME peak area (Fig. S4).

### 3.2 *In silico* toxicological and anti-inflammatory prediction

#### 3.2.1 *In silico* acute toxicity prediction via online web-servers

The *in silico* toxicity prediction with probability or confidence metrics of LME and lawsone obtained from STopTox is summarized in Table S2 (**Supplementary file)**, where red and green regions represent toxic and non-toxic fragments, respectively. STopTox predicted that lawsone was toxic via oral and dermal routes; however, LME was estimated to be non-toxic orally. The fragment maps revealed that methoxy (–OCH_3_) substitution at C–2 of lawsone attenuated its inherent reactivity. Nonetheless, LME was flagged to exhibit acute inhalational (toxicophore: methoxy and carbonyl groups) and dermal toxicities (toxicophore: entire scaffold). On the other hand, ADMETlab 3.0 classified both LME and lawsone as moderately toxic (Table S3), predicting high *in silico* carcinogenicity, moderate hepatotoxicity, and no nephro- or immunotoxicity. Our experimental/*in vivo* results (described later in this manuscript) also revealed the moderate hepatotoxicity profile of both the NQs in Wistar rats and indicating a higher toxic potential of lawsone compared to LME.

#### 3.2.2 Molecular docking analysis

Molecular docking is a quick, effective, and low-cost technique for rationalizing ligand–protein interactions and performing structure-based drug design [56]. Herein, molecular docking analysis was performed to evaluate the inhibitory interactions of LME and lawsone with redox-inflammatory targets, particularly COX-1, LOX, NAD(P)H oxidase, and MPO. As shown in Fig. 2 and Table S4, LME exhibited more favorable docking scores against COX-1 (−5.9 kcal/mol) and LOX (−6.6 kcal/mol) than lawsone (−5.3 and −5.8 kcal/mol, respectively), aspirin (−5.6 kcal/mol), and zileuton (−6.4 kcal/mol). Likewise, the docking scores of LME for NAD(P)H oxidase (−5.6 kcal/mol) and MPO (−4.7 kcal/mol) were higher than those of lawsone (−4.7 and −4.4 kcal/mol, respectively), but slightly lower than resveratrol (−6.1 and −5.1 kcal/mol, respectively). The observed LME–COX-1, LME–LOX, LME–NAD(P)H oxidase, and LME–MPO interactions mainly comprised of hydrogen bonds, van der Waals forces, amide-Pi stacking, Pi-alkyl bonding, and alkyl interactions. These findings highlight the inhibitory potential of LME against key redox-inflammatory enzymes, warranting further evaluation in redox-inflammatory disease models.

**Fig. 2:**
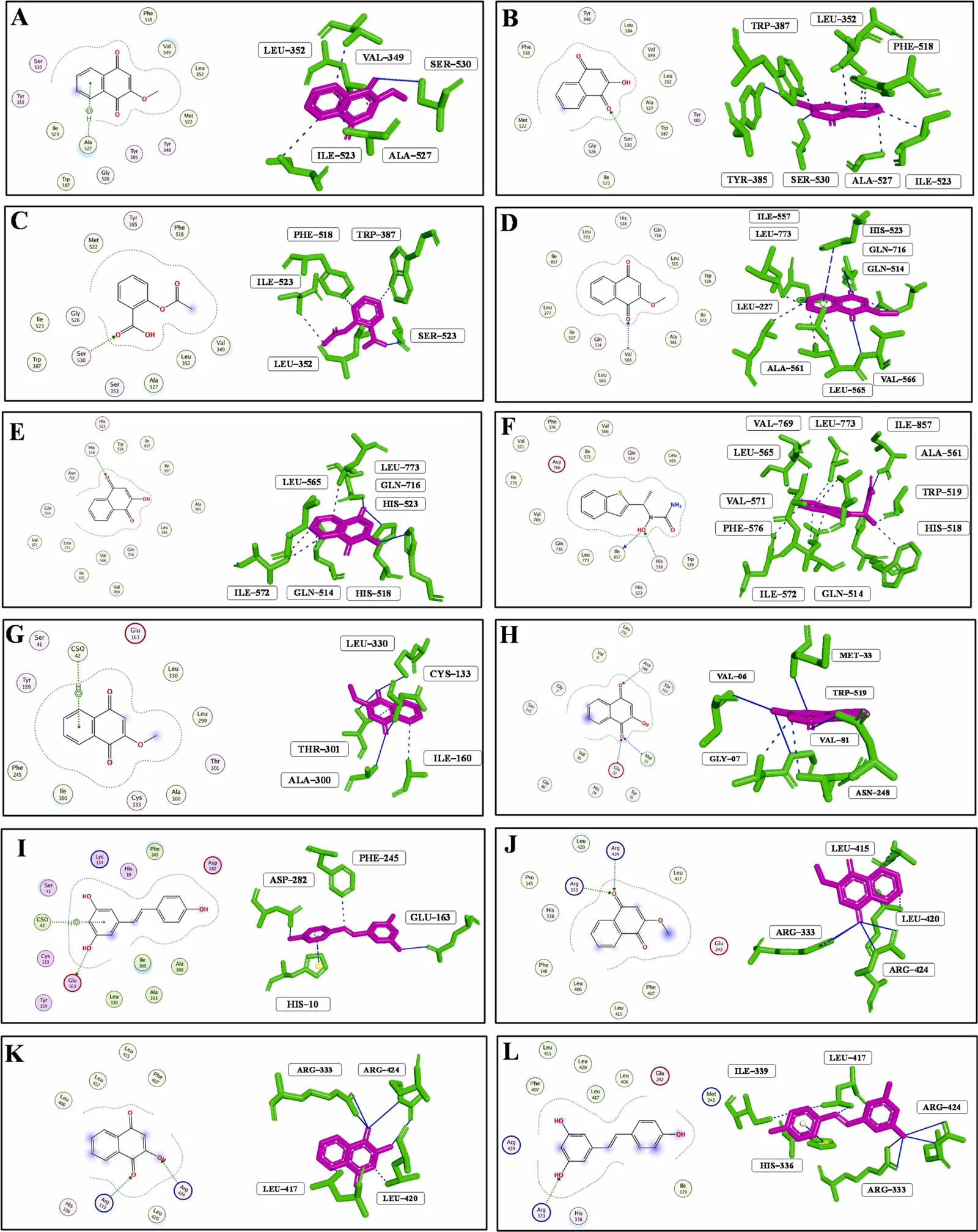
2D and 3D molecular docking interactions of (**A**) LME–COX-1, (**B**) Lawsone–COX-1, (**C**) Aspirin–COX-1, (**D**) LME–LOX, (**E**) Lawsone–LOX, (**F**) Zileuton–LOX, (**G**) LME–NAD(P)H, (**H**) Lawsone-NAD(P)H, (**I**) Resveratrol–NAD(P)H, (**J**) LME–MPO, (**K**) Lawsone–MPO, and (**L**) Resveratrol–MPO.

#### 3.2.3 MD simulations

MD simulations provide a critical insight into the structural stability and dynamic behavior of complex biological systems [57]. The structural stability of protein–ligand complexes was investigated via RMSD analysis performed over a 100 ns MD trajectory. As shown in Fig. 3**A–D,** the complexation of LME stabilized the protein frameworks, specifically during the last 40 ns of the simulations. The average RMSD values for 1DNU–LME and 2CDU–LME were lower than their corresponding apo forms (Table S5), suggesting enhanced structural stability upon ligand binding. However, the average RMSDs of 3KK6–LME and 1N8Q–LME were comparable to or higher than those of their corresponding apo forms, suggesting favorable accommodation of LME within the protein active sites.

**Fig. 3:**
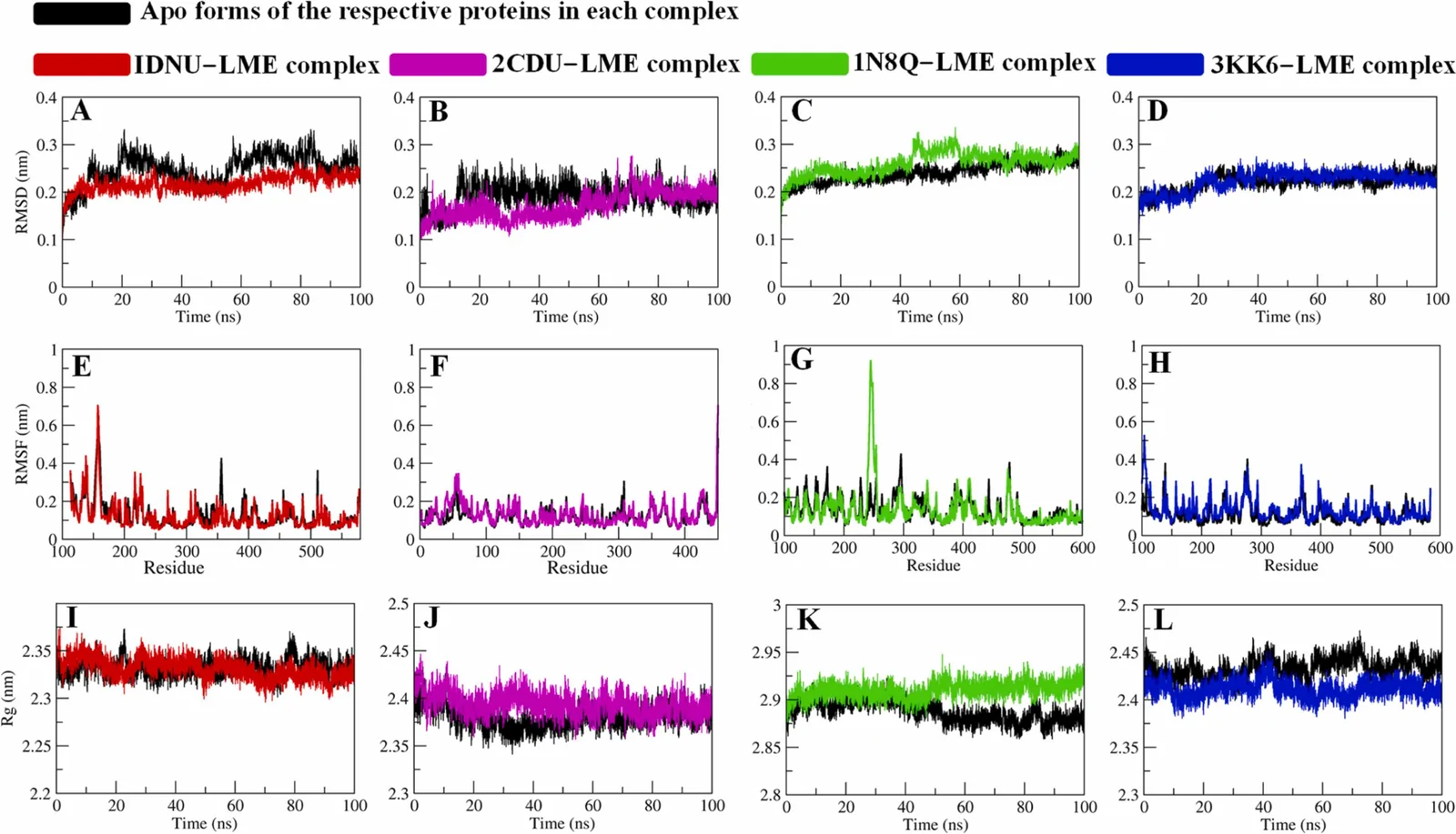
Molecular dynamics trajectories of LME–protein complexes and the corresponding apo proteins. (**A–D**) RMSD, (**E–H**) RMSF, and (**I–L**) Rg plots of LME complexed with COX-1, LOX, NAD(P)H oxidase, and MPO, compared with their respective apo forms.

RMSF analysis was performed to assess residue-level flexibility of a target protein upon ligand binding. As shown in Fig. 3**E–H** and Table S5, the complexation of LME with the protein targets induced fluctuations either lower than or comparable to their respective apo forms (except the 1N8Q–LME complex). Notably, the higher fluctuations observed in the terminal portions or loop segments of the complexes were inherently flexible, therefore, could not be actively involved in ligand binding. Furthermore, within each complex, residues around the binding site demonstrated lower RMSF values, confirming ligand-induced stabilization of these functionally important regions. Overall, the binding of LME preserved the structural rigidity of the respective proteins, validating the binding stability observed in RMSD analysis.

The Rg was studied to measure the overall compactness of protein–ligand complexes, as shown in Fig. 3**I–L**. Based on the Rg profiles, 1DNU–LME and 3KK6–LME complexes showcased lower Rg values, when compared to the respective apo forms, indicating a higher structural compactness upon LME binding. However, the 1N8Q–LME and 2CDU–LME complexes exhibited Rg values slightly lower than their corresponding apo forms, indicating minor alterations in compactness without a significant loss of structural integrity. Importantly, all the four complexes displayed slight fluctuations in Rg across the simulation, indicating the absence of major unfolding events. In a nutshell, the Rg analysis was consistent with the RMSD and RMSF findings, indicating stability, residual-level flexibility, and compactness of the protein-ligand complexes throughout the simulation period.

#### 3.2.4 DFT calculations

Fig. 4 illustrates the spatial distributions of the frontier molecular orbitals (HOMO and LUMO) along with the corresponding HOMO–LUMO energy gap for LME and lawsone. The calculated energy gap for both the NQs was approximately 10 eV, revealing a higher level of electronic stability. In general, a wide HOMO–LUMO energy gap indicates chemical inactivity or kinetic stability, because, a large amount of energy is required to excite the electrons from the HOMO (valence-like state) to the LUMO (conduction-like state). Accordingly, LME as well as lawsone exhibited resistance to electronic excitation and charge transfer under normal conditions. This energy gap underscores the structural inertness and low susceptibility of LME and lawsone to spontaneous electron donation or acceptance, contributing to their physicochemical stability.

**Fig. 4:**
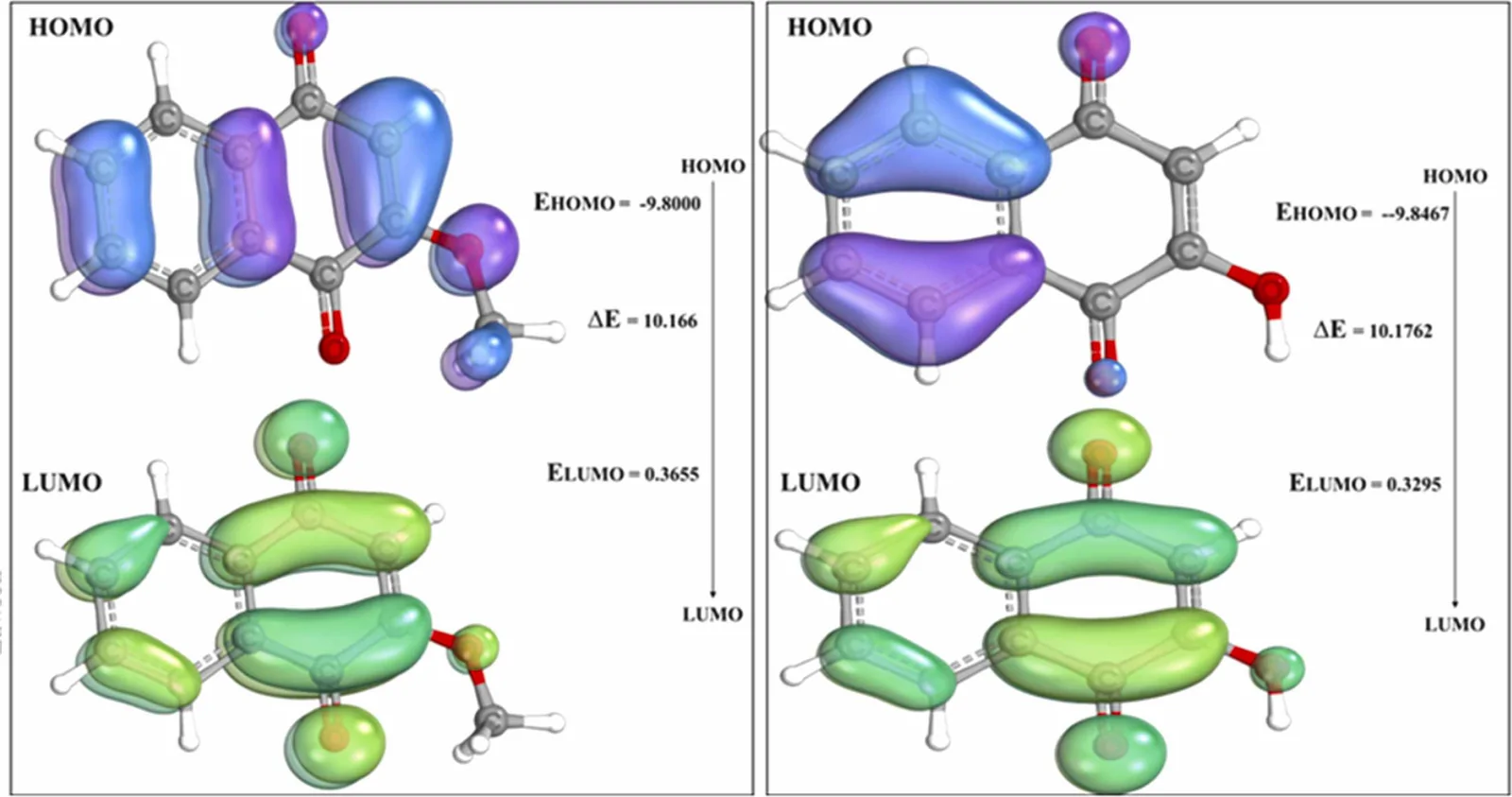
DFT-computed spatial distributions and energy levels of the highest occupied molecular orbital (HOMO) and lowest unoccupied molecular orbital (LUMO) of (**A**) LME and (**B**) lawsone.

### 3.3 *In vitro* COX-1 and COX-2 inhibitory activities

Table 1 summarizes the IC_50_ values of LME, lawsone, indomethacin, and celecoxib against COX-1 and COX-2. These IC_50_ values were derived from the dose-response curves (Fig. 5**A** and **B**) generated using eight graded concentrations (0.19–25 µM), with the corresponding 95% confidence intervals (CIs) reported for each estimate. The results of the COX-1 inhibitory assay revealed 8.1 µM as an IC_50_ value of LME, surpassing lawsone (IC_50_: 14.9 µM) and celecoxib (IC_50_: 16.4 µM), however, it was less potent than that of indomethacin (0.78 µM). Likewise, for COX-2 inhibition, the IC_50_ value for LME was 4.2 µM, lower than that of lawsone (IC_50_ = 9.2 µM). Nonetheless, its inhibitory activity remained low when compared to celecoxib (IC_50_ = 0.59 µM) and indomethacin (IC_50_ = 1.3 µM).

**Table 1:** *In vitro* inhibitory activities of LME and lawsone against COX-1 and COX-2 enzymes.

Compound	IC_50_ (µM)^a^		SI^b^ (COX-1/COX-2)
	COX-1	COX-2	
LME	8.1 ± 0.3	4.2 ± 0.2	1.9
Lawsone	14.9 ± 0.6	9.2 ± 0.3	1.6
Indomethacin	0.78 ± 0.04	1.3 ± 0.05	0.6
Celecoxib	16.4 ± 0.	0.59 ± 0.03	27.9

a Data are expressed as IC_50_ values (Mean ± SD) of experiments in triplicate (*p* ≤ 0.05)

b Experimental COX-2 selectivity index (COX-1/COX-2)

**Fig. 5:**
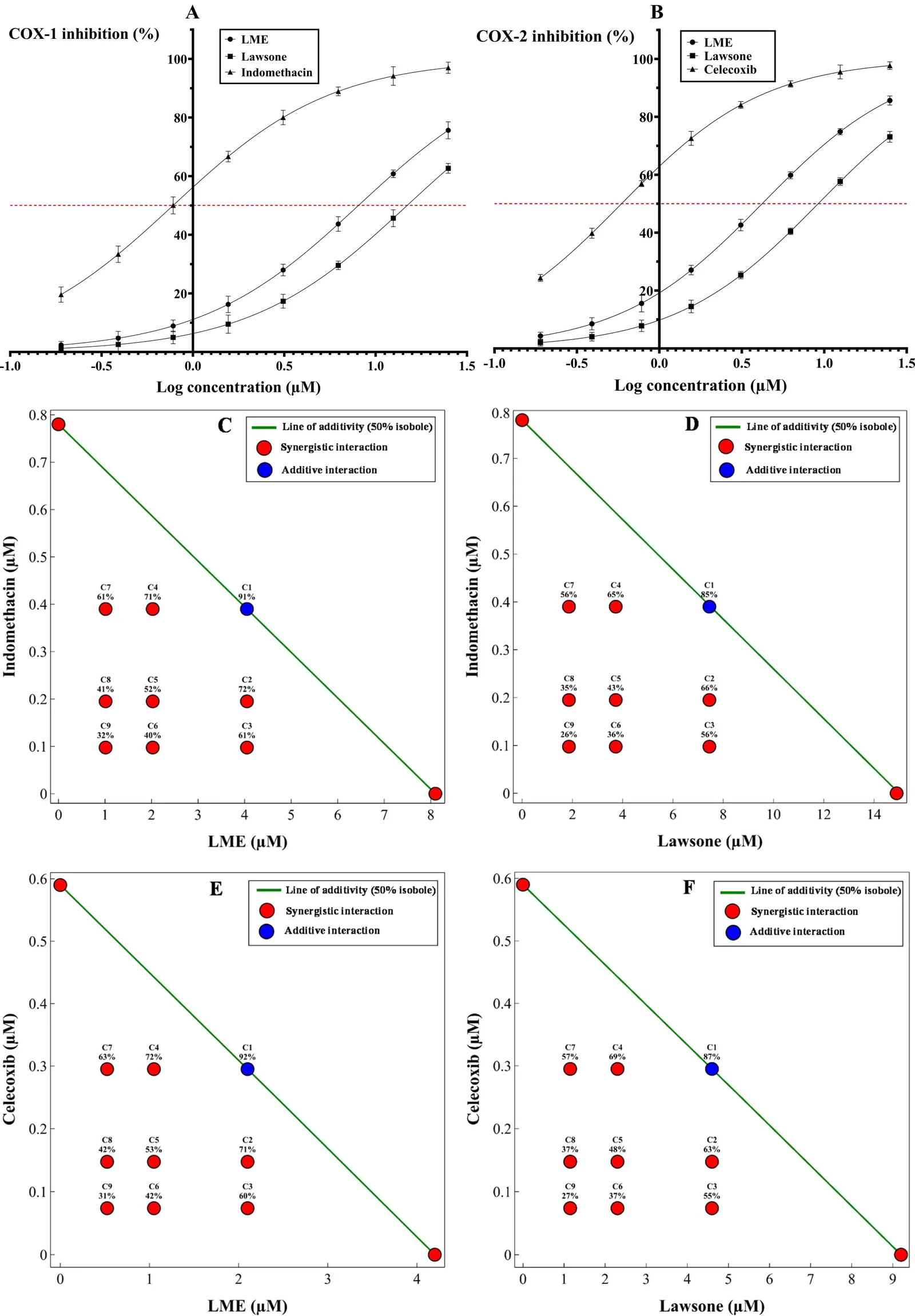
Log dose–response curves for LME and lawsone against (**A**) COX-1 and (**B**) COX-2 with corresponding IC_50_ values, exhibiting 95% confidence intervals. Data are expressed as mean ± SD (*n* = 3). Isobolograms representing the interactions of (**C**) LME–indomethacin against COX-1, (**D**) Lawsone–indomethacin against COX-1, (**E**) LME–celecoxib against COX-2, and (**F**) Lawsone–celecoxib against COX-2 at the 50% inhibitory level. The line connecting the individual IC_50_ values represents additivity. The blue marker indicates an additive combination on the isobole, whereas red markers indicate synergistic combinations below the isobole. C1–C9 represent the nine combinations of a naphthoquinone (LME or lawsone) with indomethacin or celecoxib. Numerical labels indicate the observed mean percentage inhibition at each combination level.

In the present study, LME inhibited COX-2 slightly higher than COX-1, with a selectivity index (SI) of 1.9, indicating a dual COX inhibitory profile with limited COX-2 preference. Although LME could not showcase being a true COX-2-selective inhibitor, a relative shift toward COX-2 inhibition might still be clinically favorable. Because COX-2 selective inhibition exerts lesser gastrotoxicity and platelet aggregation, when compared to purely nonselective COX inhibitors [58]. Nonetheless, given the limited selectivity index of LME, this potential advantage should be interpreted cautiously and warrants further validation. These *in vitro* results are consistent with our *in silico* findings, wherein LME inhibited COX more potently than lawsone. Previously, LME was reported to exhibit immunomodulatory activities [16,17], warranting its further investigation as a potential anti-inflammatory lead compound.

### 3.4 Inhibitory interactions of LME/lawsone combined with indomethacin and celecoxib

The inhibitory interactions of LME and lawsone, combined with indomethacin or celecoxib, against COX-1 and COX-2 were evaluated using isobolographic analysis (Fig. 5**C–F** and Table 2, Table 3). Based on isobolographic criteria, the combination of ½IC_50_ LME/lawsone with ½IC_50_ indomethacin or celecoxib was classified as additive. However, ½IC_50_ or ¼IC_50_ LME combined with ½IC_50_ or ¼IC_50_ of indomethacin/celecoxib fell below the line of additivity (attaining 50% inhibition), therefore, were termed as synergistic. Similar results (synergism) were observed when ⅛IC_50_ of LME was combined with ½IC_50_ of indomethacin or celecoxib. The remaining combinations of LME–indomethacin or LME–celecoxib were deemed inconclusive, as they failed to reach a 50% inhibitory effect. Likewise, the combinations of ½IC_50_ lawsone with ½IC_50_ or ¼IC_50_ indomethacin/celecoxib were found to be synergistic. Similar results (synergism) were obtained, when ¼IC_50_ or ⅛IC_50_ of lawsone was combined with ½IC_50_ indomethacin/celecoxib. The remaining combinations of lawsone–indomethacin or lawsone–celecoxib were deemed inconclusive, as they failed to reach a 50% inhibitory effect level. Notably, none of the interactions of LME or lawsone with the standard drugs were found to be antagonistic.

**Table 2:** Inhibitory interactions of LME or lawsone combined with indomethacin against COX-1 and their 50% isobolographic interpretation.

Compound–IC_50_	Indomethacin–IC_50_	% Inhibition^a^		% Inhibition^c^	Position on the isobologram	Interpretation
		Naphthoquinone	Indomethacin			
LME–(½IC_50_)	Indomethacin–(½IC_50_)	22.3 ± 1.2	23.1 ± 1.1	90.8 ± 4.8	On the 50% isobole	Additive
	Indomethacin–(¼IC_50_)	22.3 ± 1.2	12.9 ± 0.4	72.3 ± 3.2	Below the line	Synergistic
	Indomethacin–(⅛IC_50_)	22.3 ± 1.2	7.8 ± 0.1	61.5 ± 2.9	Below the line	Synergistic
LME–(¼IC_50_)	Indomethacin–(½IC_50_)	12.2 ± 0.5	23.1 ± 1.1	70.9 ± 4.3	Below the line	Synergistic
	Indomethacin–(¼IC_50_)	12.2 ± 0.5	12.9 ± 0.4	52.3 ± 2.8	Below the line	Synergistic
	Indomethacin–(⅛IC_50_)	12.2 ± 0.5	7.8 ± 0.1	40.5 ± 2.1	Below the line	Inconclusive*
LME–(⅛IC_50_)	Indomethacin–(½IC_50_)	7.6 ± 0.2	23.1 ± 1.1	61.1 ± 3.1	Below the line	Synergistic
	Indomethacin–(¼IC_50_)	7.6 ± 0.2	12.9 ± 0.4	41.2 ± 2.3	Below the line	Inconclusive*
	Indomethacin–(⅛IC_50_)	7.6 ± 0.2	7.8 ± 0.1	31.8 ± 1.7	Below the line	Inconclusive*
Lawsone–(½IC_50_)	Indomethacin–(½IC_50_)	22.9 ± 1.1	23.1 ± 1.1	85.4 ± 4.1	On the 50% isobole	Additive
	Indomethacin–(¼IC_50_)	22.9 ± 1.1	12.9 ± 0.4	66.3 ± 3.4	Below the line	Synergistic
	Indomethacin–(⅛IC_50_)	22.9 ± 1.1	7.8 ± 0.1	55.9 ± 2.9	Below the line	Synergistic
Lawsone–(¼IC_50_)	Indomethacin–(½IC_50_)	11.8 ± 0.5	23.1 ± 1.1	65.5 ± 3.1	Below the line	Synergistic
	Indomethacin–(¼IC_50_)	11.8 ± 0.5	12.9 ± 0.4	43.5 ± 2.5	Below the line	Inconclusive*
	Indomethacin–(⅛IC_50_)	11.8 ± 0.5	7.8 ± 0.1	36.1 ± 1.9	Below the line	Inconclusive*
Lawsone–(⅛IC_50_)	Indomethacin–(½IC_50_)	5.9 ± 0.2	23.1 ± 1.1	56.3 ± 2.8	Below the line	Synergistic
	Indomethacin–(¼IC_50_)	5.9 ± 0.2	12.9 ± 0.4	35.1 ± 1.9	Below the line	Inconclusive*
	Indomethacin–(⅛IC_50_)	5.9 ± 0.2	7.8 ± 0.1	26.4 ± 1.2	Below the line	Inconclusive*

% Inhibition^a^: % Inhibition by LME, lawsone or indomethacin alone, % Inhibition^c^: % Inhibition by LME/lawsone combined with indomethacin, *Inconclusive: The combination exhibited less than 50% enzyme inhibition, therefore, could not be classified reliably on a 50% isobologram. Data are expressed as IC_50_ values (Mean ± SD) of experiments in triplicate (*p* ≤ 0.05)

**Table 3:** Inhibitory interactions of LME or lawsone combined with celecoxib against COX-2 and their 50% isobolographic interpretation.

Compound–IC_50_	Celecoxib–IC_50_	% Inhibition^a^		% Inhibition^c^	Position on the isobologram	Interpretation
		Naphthoquinone	Celecoxib			
LME–(½IC_50_)	Celecoxib–(½IC_50_)	21.9 ± 1.3	23.8 ± 0.9	91.7 ± 4.9	On the 50% isobole	Additive
	Celecoxib–(¼IC_50_)	21.9 ± 1.3	12.9 ± 0.7	71.1 ± 3.1	Below the line	Synergistic
	Celecoxib–(⅛IC_50_)	21.9 ± 1.3	7.9 ± 0.1	59.9 ± 3.1	Below the line	Synergistic
LME–(¼IC_50_)	Celecoxib–(½IC_50_)	12.6 ± 0.4	23.8 ± 0.9	71.7 ± 3.8	Below the line	Synergistic
	Celecoxib–(¼IC_50_)	12.6 ± 0.4	12.9 ± 0.7	52.9 ± 2.7	Below the line	Synergistic
	Celecoxib–(⅛IC_50_)	12.6 ± 0.4	7.9 ± 0.1	41.6 ± 2.1	Below the line	Inconclusive*
LME–(⅛IC_50_)	Celecoxib–(½IC_50_)	7.2 ± 0.2	23.8 ± 0.9	63.2 ± 3.5	Below the line	Synergistic
	Celecoxib–(¼IC_50_)	7.2 ± 0.2	12.9 ± 0.7	41.6 ± 1.8	Below the line	Inconclusive*
	Celecoxib–(⅛IC_50_)	7.2 ± 0.2	7.9 ± 0.1	31.4 ± 1.2	Below the line	Inconclusive*
Lawsone–(½IC_50_)	Celecoxib–(½IC_50_)	21.2 ± 1.2	23.8 ± 0.9	86.8 ± 4.1	On the 50% isobole	Additive
	Celecoxib–(¼IC_50_)	21.2 ± 1.2	12.9 ± 0.7	63.5 ± 3.2	Below the line	Synergistic
	Celecoxib–(⅛IC_50_)	21.2 ± 1.2	7.9 ± 0.1	54.6 ± 2.8	Below the line	Synergistic
Lawsone–(¼IC_50_)	Celecoxib–(½IC_50_)	12.1 ± 0.6	23.8 ± 0.9	68.8 ± 3.2	Below the line	Synergistic
	Celecoxib–(¼IC_50_)	12.1 ± 0.6	12.9 ± 0.7	47.6 ± 2.2	Below the line	Inconclusive*
	Celecoxib–(⅛IC_50_)	12.1 ± 0.6	7.9 ± 0.1	37.4 ± 1.9	Below the line	Inconclusive*
Lawsone–(⅛IC_50_)	Celecoxib–(½IC_50_)	6.3 ± 0.3	23.8 ± 0.9	56.9 ± 3.1	Below the line	Synergistic
	Celecoxib–(¼IC_50_)	6.3 ± 0.3	12.9 ± 0.7	37.3 ± 1.8	Below the line	Inconclusive*
	Celecoxib–(⅛IC_50_)	6.3 ± 0.3	7.9 ± 0.1	26.8 ± 0.9	Below the line	Inconclusive*

% Inhibition^a^: % Inhibition by LME, lawsone or celecoxib alone, % Inhibition^c^: % Inhibition by LME/lawsone combined with celecoxib, *Inconclusive: The combination exhibited less than 50% enzyme inhibition, therefore, could not be classified reliably on a 50% isobologram. Data are expressed as IC_50_ values (Mean ± SD) of experiments in triplicate (*p* ≤ 0.05).

For an adult, clinically prescribed dose of indomethacin is 25 mg three times a day [59], however, celecoxib is prescribed as 100 mg twice a day [60]. Both the medications are generally well tolerated, however, their prolonged administration or accidental overdosing is associated with a number of lethal side effects. Indomethacin was reported to induce stroke, cardiomyopathy, gastrointestinal bleeding, hepatorenal toxicity, and hypersensitivity [59]. Conversely, celecoxib was found to be linked with infertility, gastrointestinal toxicity, myocardial infarction, renal impairment, asthmatic exacerbation, and acute hypersensitivity [61].

The aforementioned side effects have prompted the clinicians to trim the standard doses of indomethacin or celecoxib, diminishing their therapeutic efficacy. This led us to evaluate the potential interactions of LME and lawsone, when combined with indomethacin and celecoxib against COX-1/2. The results uncovered that combining these agents at lower doses achieved higher enzyme suppression, which might lead to a decreased burden of adverse effects. The observed synergistic interactions between a NQ (LME or lawsone) and a standard drug (indomethacin or celecoxib) can be attributed to their distinct molecular structures and sizes. Differences in molecular bulks can reduce steric hindrances, facilitating an efficient binding of LME or lawsone to COX enzymes. Similar behavior has been previously reported, wherein LME combined with acarbose exhibited synergistic inhibitory activity against α-glucosidase [13].

### 3.5 *In vitro* hemocompatibility assessment

*In vitro* hemocompatibility assessment is crucial for determining the impact of a lead compound on human RBCs, minimizing the need for *in vivo* experiments during preliminary screening [62]. *In vitro* hemolytic activities of LME and lawsone are depicted in Fig. 6. Under the tested conditions, separate incubation of human RBCs with LME or lawsone (5, 10, or 20 µM) induced low hemolysis (≤ 12%), relative to the positive control, Triton X-100 (100% hemolysis). Notably, these findings should not be interpreted as evidence of complete hemolytic safety, given that the present assay was restricted to a narrow concentration range and does not preclude hemolytic effects at higher exposures. These results are consistent with our acute and subacute toxicity findings, wherein an acute or repeated oral administration of LME and lawsone could not significantly alter erythrocyte-related hematological parameters.

**Fig. 6:**
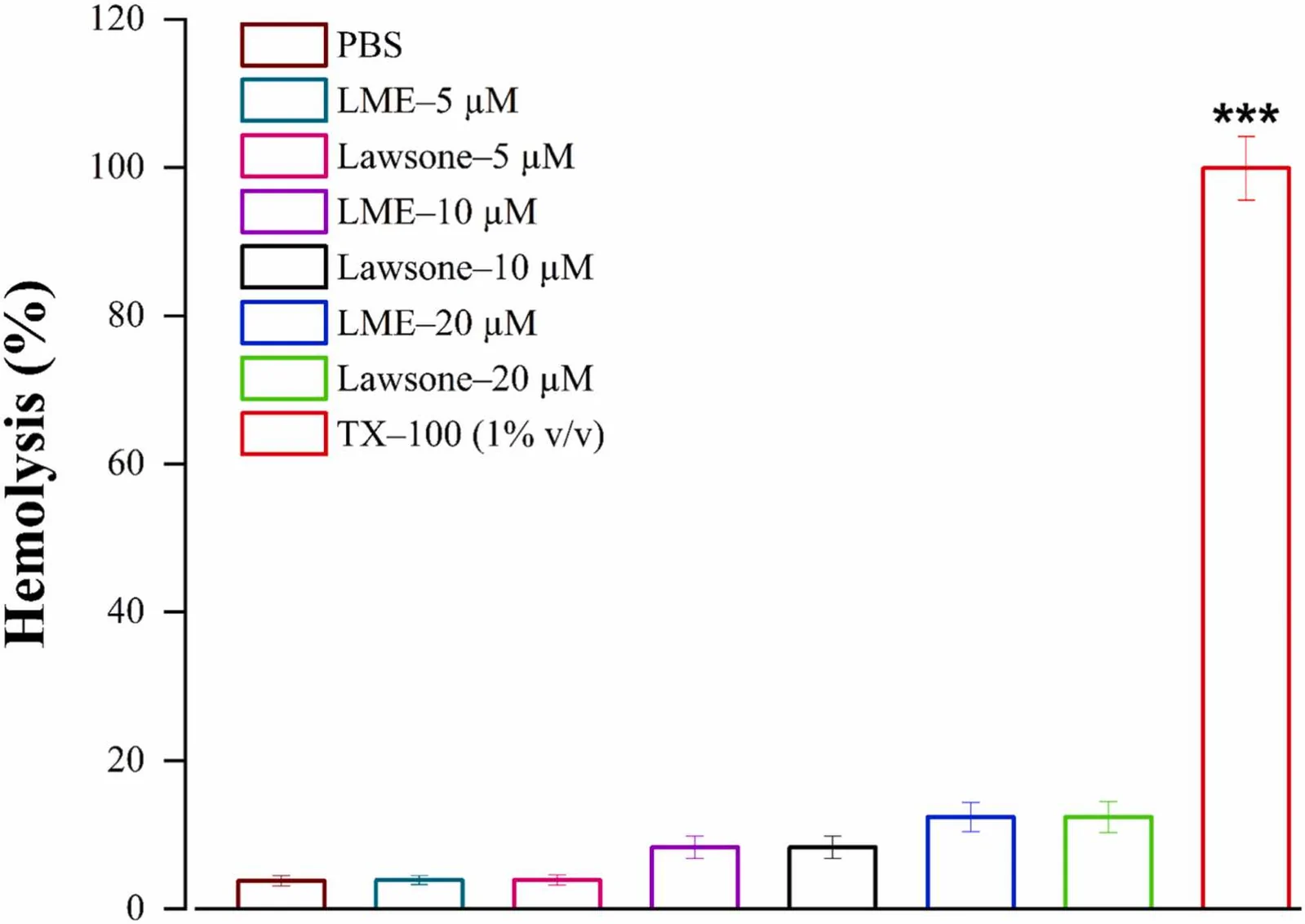
Hemolytic activity (%) of LME and lawsone (5, 10, and 20 µM) relative to Triton X-100 (1% v/v) using human RBCs. Data are presented as mean ± SD (*n* = 3 per group). Statistical significance was assessed by one-way ANOVA followed by Tukey’s post hoc test; ***p < 0.0001.

### 3.6 *In vivo* toxicity assessment

#### 3.6.1 Acute and subacute oral toxicity assessment

Acute oral toxicity testing identifies a lethal dose threshold of a lead compound, while subacute toxicity study reveals the cumulative toxicity and safe dosage range [45]. Acute oral sings of toxicity and mortality induced by LME and lawsone are summarized in Table S6**.** Based on these results, the NOAEL of 50 mg/kg was established for both the NQs, however, at 300 mg/kg, LME caused mild lethargy (33% mortality). In contrast, lawsone at an equivalent dose induced 100% mortality, preceded by tremors, respiratory distress, and paralysis. Likewise, LME at 2000mg/kg produced dyspnea, loss of reflexes, tremors, paralysis, coma, and death in all the rats. These findings align with the known mechanism of NQ-induced toxicity mediated via oxidative mitochondrial dysfunction [63]. On the other hand, in a 28-day repeated oral toxicity study, neither LME nor lawsone induced treatment-related adverse effects or mortality in male or female rats at any of the doses tested, establishing the NOAEL of at least 50 mg/kg/day (Table S7). Nonetheless, transient mild lethargy and tachycardia were observed immediately after the first dosage, which might have resulted from the stress-related adaptation [64].

#### 3.6.2 Acute oral LD_50_ calculation via Karber’s method

In acute toxicity testing, determination of LD_50_ serves as a gold standard, wherein Karber's method remains preferred due to its methodological simplicity, ease of calculation, and satisfactory accuracy. Additionally, in its preliminary phase, a small number of animals are exposed to a wide dosage range [65,66], aligning with the stepwise and limited-animal design of OECD 423. Individual mortality data, dose-sequence details, and the complete LD_50_ calculations for LME and lawsone are summarized in Table S8 and Equation S1 of the **Supplementary file**. Based on the mortality pattern observed at different dosage levels, the oral LD_50_ values for LME and lawsone were calculated to be 542 and 175 mg/kg, respectively, corresponding to the GHS acute oral toxicity categories 4 (harmful if swallowed) and 3 (toxic if swallowed) [67], indicating a wider margin of safety for LME than that of lawsone.

#### 3.6.3 Metabolic effects in male and female rats

A toxicant can adversely affect food and water intake in animal models, thereby altering their body weight gain [68], glycemic levels [69], and relative organ weight [70]. As illustrated in Fig. S5 **(A, D, G,** and **J)**, acute oral administration of 50 mg/kg LME or lawsone did not affect body weight gain, food/water intake, FBG levels, or relative organ weight in female rats. However, at 300 mg/kg, LME slightly reduced weight gain and food/water consumption compared to the controls, without any significant impact on FBG levels or relative organ weight. Conversely, a 28-day repeated oral administration of LME or lawsone (15, 30, and 50 mg/kg) produced a normal weight gain without altering food/water intake, FBG levels, or relative weights of the major organs, with no sex-related differences observed (Fig. S5B**, C, E, F, H, I, K,** and **L**).

#### 3.6.4 Effect on the functional biomarkers of liver, kidney, and heart

The liver and kidneys are sensitive to drug-induced toxicity; injury to these organs can elevate serum levels of hepatic (AST, ALT, ALP, and TB) and renal (creatinine and BUN) functional biomarkers [71,72]. As depicted in Fig. S6 **(A–F),** neither LME nor lawsone, at any dosage level, during acute or subacute exposure, significantly altered AST, ALT, ALP, TB, BUN, and creatinine levels compared to the controls, with no sex-related differences observed. Nonetheless, an acute administration of 300 mg/kg LME induced a mild elevation of hepatic biomarkers. These findings are consistent with our histopathological results. Previous studies have reported hepatoprotective and nephroprotective effects of LME and lawsone, driven by their antioxidant potentials [14,20,21]. Dyslipidemia increases the incidence and progression of cardiomyopathies [73]; therefore, estimating HDL, LDL, TC, or TGs levels in a toxicological study is imperative. Here, neither acute nor repeated oral administration of LME or lawsone, at any dosage level, significantly altered the lipid profile of rat models, compared to the control group, with no significant sex-related differences observed (Fig. S6**G–I**).

#### 3.6.5 Hematological effects

Hematological parameters serve as valuable indicators for assessing the adverse effects of a xenobiotic on immune function and oxygen-carrying capacity [74]. In this context, as shown in Fig. S7**,** neither acute nor repeated oral administration of LME or lawsone, at any dosage level, significantly altered the hematological parameters of male as well as female rats, compared to the control group, with no significant sex-related differences observed.

#### 3.6.6 Histopathological effects on the visceral organs

Histopathological assessment is an integral component of toxicity testing, offering valuable insights into cellular and tissue damage caused by toxic exposure [75]. The acute and subacute effects of LME and lawsone on the histology of the major visceral organs in rats are illustrated in Fig. 7 and Fig. S8, respectively. However, their semiquantitative histopathological severity scores [48], are summarized in Table S9. Based on acute histopathological findings, the heart, liver, lungs, kidneys, and splenic tissues from rats receiving 50 mg/kg of LME or lawsone revealed normal histology, with no vascular or inflammatory alterations, as compared to the control group. However, at the higher doses of LME (300 and 2000mg/kg) or lawsone (300 mg/kg), the diameter and appearance of central vein, portal veins, and hepatic sinusoids revealed abnormal histology, showing moderate hepatitis. Spleen histology showed no signs of toxicity at the lower doses tested, whereas mild decellularization was observed at the highest doses (2000mg/kg LME and 300 mg/kg lawsone), indicating a moderate adverse effect on splenic architecture. On the other hand, subacute histopathological effects of LME or lawsone (15, 30, and 50 mg/kg) demonstrated normal histology of the liver, heart, lungs, kidneys, and spleen of male and female rats, with no sex-related differences observed. In conclusion, all the visceral organs maintained a normal histology at least at 50 mg/kg of LME or lawsone in acute as well as subacute toxicity assessments. Notably, the histopathological findings were found consistent with the relative organ weights and biochemical results of our study.

**Fig. 7:**
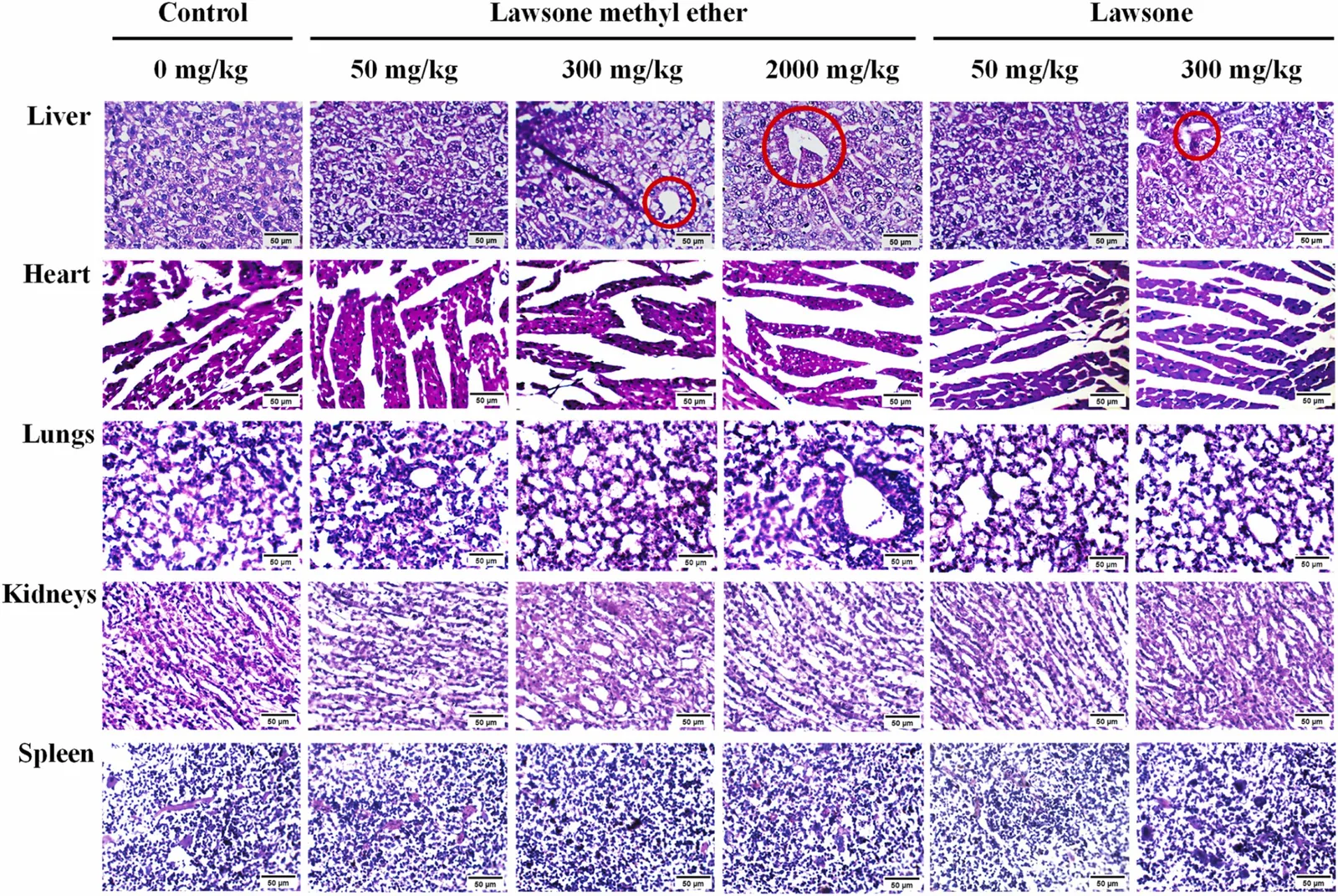
Representative photomicrographs of hematoxylin and eosin (H&E)-stained sections of the liver, heart, lungs, kidneys, and spleen from female rats in the control and acute-treatment groups receiving single oral doses of LME (50, 300, or 2000mg/kg) or lawsone (50 or 300 mg/kg). Red circles indicate histopathological lesions. Scale bar = 50 µm.

#### 3.6.7 *In vivo* redox-modulatory activities of LME and lawsone

The antioxidant effects of LME and lawsone on TBARS, GSH, SOD, and CAT within plasma and different tissue homogenates of rats are shown in Fig. 8**.** According to the results, a repeated oral administration of LME and lawsone at 30 and 50 mg/kg for 28 days demonstrated a significant (*p* < 0.05) antioxidant activity, reflected by decreased TBARS and increased GSH, SOD, and CAT levels relative to the controls in male and female rats, exhibiting no sex-related significant differences (the data only shown for 50 mg/kg in the Fig. 8). Conversely, acute oral administration of 50 mg/kg of LME or lawsone showed no significant effect on the redox state of female rats. Nonetheless, a significant (*p* < 0.05) antioxidant response was observed in female rats administered with 300 mg/kg of acute oral LME, Notably, this dose was also associated with mortality (1/3), therefore, the observed redox changes may not be considered beneficial. Alternatively, they might reflect an acute stress, compensatory response, or a pro-oxidant effect of LME preceding mortality.

**Fig. 8:**
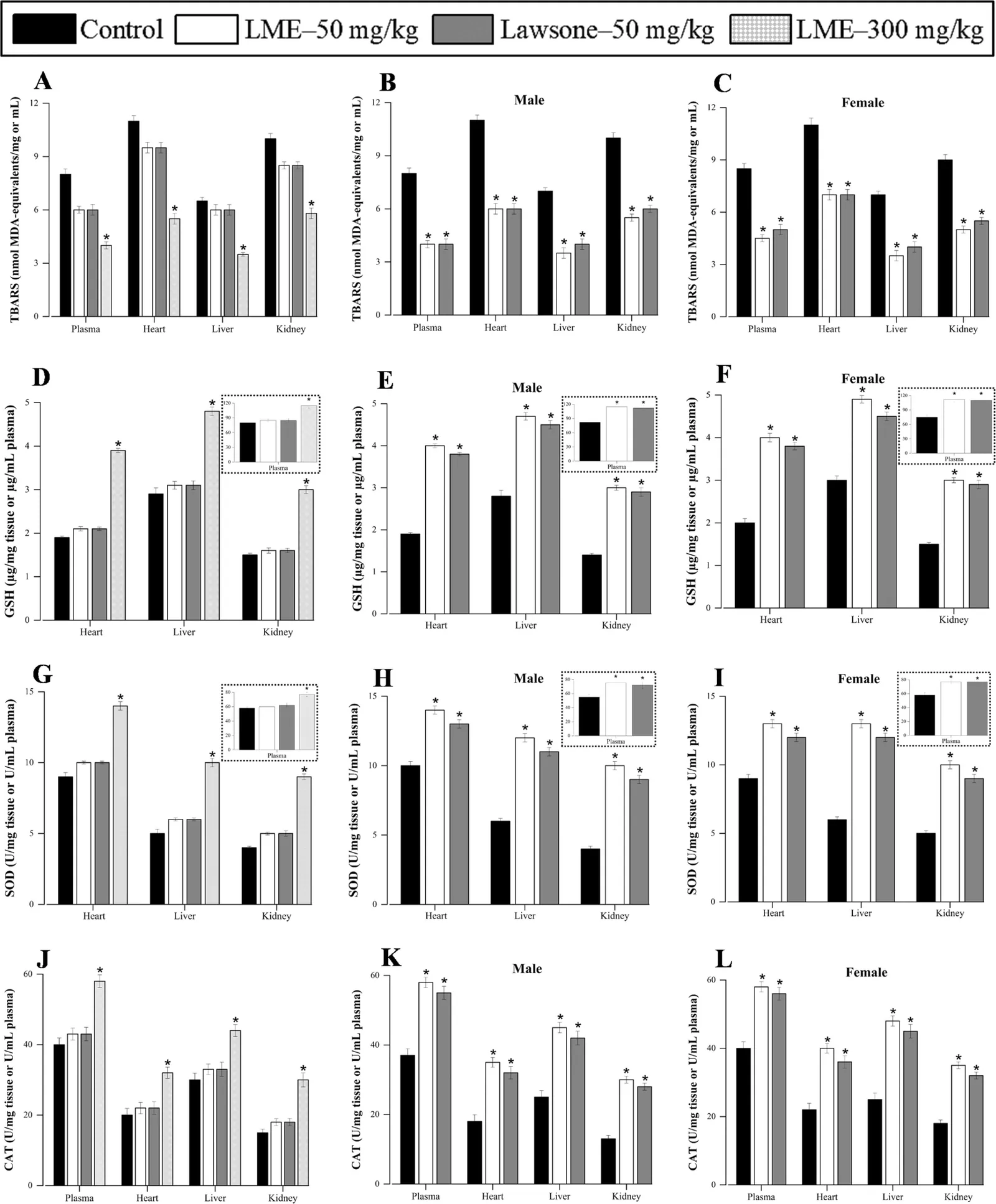
Acute effects of LME and lawsone on (**A**) TBARS, (**D**) GSH, (**G**) SOD, and (**J**) CAT in female rats. Statistical significance was determined using one-way ANOVA followed by Tukey's post-hoc test (*p < 0.05). Subacute effects of LME and lawsone on (**B** and **C**) TBARS, (**E** and **F**) GSH, (**H** and **I**) SOD, and (**K** and **L**) CAT in male and female rats, respectively. Statistical significance was determined using two-way ANOVA (Treatment × Sex), followed by Tukey's post-hoc test (*p < 0.05)*.* No statistically significant sex related differences were observed, therefore, the data for male and female rats were presented separately to improve the clarity.

Based on the aforementioned findings, the observed antioxidant activities of LME or lawsone may be linked to their underlying anti-inflammatory effect. Nonetheless, changes in redox markers using healthy animals do not, by themselves, demonstrate organ protection or anti-inflammatory efficacy. Thereby, these results should be considered preliminary rather than conclusive. These findings are consistent with our molecular docking and molecular dynamics predictions, showcasing favorable interactions of LME with LOX, NAD(P)H oxidase, and MPO. Previously, LME [14] and lawsone [76,77] were reported to exhibit antioxidant potentials, further corroborating the redox-modulatory effects observed in the current study. A schematic representation of the COX-inhibitory and redox-modulatory effects of LME and lawsone is illustrated in Fig. 9.

**Fig. 9:**
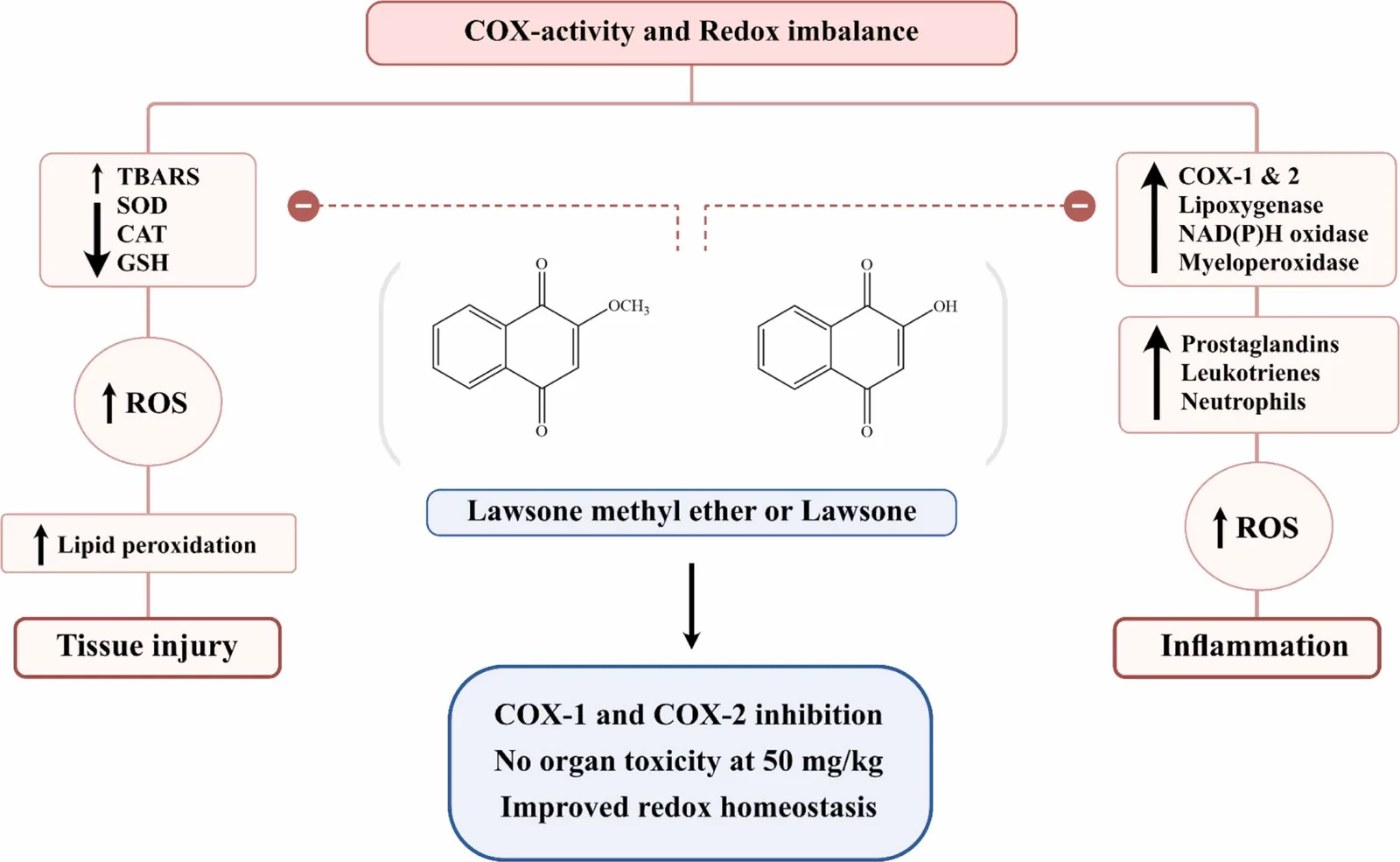
Integrated mechanistic overview elaborating the COX-inhibitory and redox-modulatory effects of LME and lawsone.

The current study exhibits a few limitations. Heme and other relevant cofactors were removed from the target proteins prior to molecular docking, which may have affected the native binding environment of the redox-inflammatory protein targets. Moreover, COX-2 was not included in the docking and molecular dynamics analyses despite its experimentally observed inhibition and MD simulations were run as a single, non-replicated trajectory due to time and resource constraints. Therefore, future studies should investigate LME/lawsone–COX-2 interactions using cofactor-retained protein models and involve multiple replicates of MD trajectories to confirm their reproducibility. Additionally, the TBARS assay is not specific to malondialdehyde (MDA) and may be influenced by other TBA-reactive substances. Thereby, future studies should employ HPLC or LC-MS/MS analysis to validate the impact of LME/lawsone specifically on MDA levels. In the subacute oral toxicity study, 50 mg/kg/day was the maximum dose tested; therefore, future chronic toxicity studies should evaluate doses exceeding 50 mg/kg/day to determine the maximum NOAEL threshold for LME and lawsone. Finally, acute and subacute toxicity testing alone should not be considered conclusive; chronic toxicity assessment of LME is essential to establish its long-term safety and clinical applicability.

## 4 Conclusions

Findings of the current study demonstrated the COX-inhibitory, redox-modulatory, and oral toxicity potentials of LME and lawsone via an integrated computational, enzyme-inhibitory, and *in vivo* approach. At the computational level, molecular docking analysis revealed favorable docking scores/interactions between LME and key redox-inflammatory targets, further substantiated by MD simulations and DFT calculations. Consistent with these predictions, *in vitro* assays showcased COX-1 and COX-2 inhibition by LME, displaying synergistic isobolographic interactions, when combined with indomethacin or celecoxib. *In vivo* oral administration of LME demonstrated redox-modulatory potential by ameliorating GSH, SOD, CAT, and TBARS levels, with efficacy higher than lawsone. However, the NOAEL for LME and lawsone was at least 50 mg/kg/day under the conditions of 28 days subacute toxicity study. Overall, LME was better tolerated than lawsone, showing *in vitro* COX inhibitory and *in vivo* redox-modulatory effects. Nonetheless, chronic toxicity assessment and comprehensive mechanistic elucidation are essential to validate its long-term safety and therapeutic applicability in redox-inflammatory pathologies.

## Funding

This research was financially supported by the Ph.D. Award 2020–2022 from the Discipline of Excellence, Faculty of Pharmaceutical Sciences, Prince of Songkla University, Thailand. Additionally, a Research Grant for the Thesis in the Fiscal Year 2022 was allocated by the Graduate School, Prince of Songkla University, Thailand.

## CRediT authorship contribution statement

**Muhammad Khan:** Writing – original draft, Methodology, Investigation, Formal analysis, Data curation, Conceptualization. **Asad Ur Rahman:** Writing – original draft, Methodology, Investigation, Formal analysis, Conceptualization. **Phisit Khemawoot:** Writing – review & editing, Supervision, Resources, Project administration, Methodology, Formal analysis. **Muhammad Ajmal Shah:** Investigation, Data curation. **Zaheer Ul-Haq:** Investigation, Data curation. **Nadeem Ahmad:** Investigation, Data curation. **Tehmina Sohail:** Investigation, Data curation. **Pharkphoom Panichayupakaranant:** Writing – review & editing, Supervision, Resources, Project administration, Methodology, Funding acquisition, Formal analysis.

## Declaration of Generative AI and AI-assisted technologies in the writing process

During the preparation of this work, the authors used Claude Sonnet 5 (Anthropic) solely to improve the manuscript's clarity, readability, and English-language expression. The authors subsequently reviewed, verified, and edited all AI-assisted content and take full responsibility for the published article. No AI tool was used to generate or interpret the study data, results, figures, or scientific conclusions.

## Declaration of Competing Interest

The authors declare that they have no known competing financial interests or personal relationships that could have appeared to influence the work reported in this paper.

## Data Availability

Data will be made available on request.
