# Supplementary material for: Effects of lawsone and lawsone methyl ether on cyclooxygenase activity, redox balance, and organ-specific toxicity: An integrated computational and experimental study
Source: Toxicol Rep. 2026 Sep 22;17:102345. doi: 10.1016/j.toxrep.2026.102345 (PMC13635553; doi:10.1016/j.toxrep.2026.102345)
Supplement: Supplementary file 1 [file mmc1.docx]

**Effects of lawsone and lawsone methyl ether on cyclooxygenase activity, redox balance, and organ-specific toxicity: An integrated computational and experimental study**

Muhammad Khan^a,†^, Asad Ur Rahman^b,†^, Phisit Khemawoot^b*^, Muhammad Ajmal Shah^c^, Zaheer Ul-Haq^d^, Nadeem Ahmad^d^, Tehmina Sohail^e^, and Pharkphoom Panichayupakaranant^f,g*^

^a^ *Department of Pharmacology, Faculty of Pharmacy, Federal Urdu University of Arts, Science, and Technology, Karachi 75300, Pakistan*

^b^ *Chakri Naruebodindra Medical Institute, Faculty of Medicine Ramathibodi Hospital, Mahidol University, Samut Prakan, Thailand*

^c^ *Department of Pharmacy, Hazara University, Mansehra 21300, Pakistan*

^d^ *Dr. Panjwani Center for Molecular Medicine and Drug Research, International Center for Chemical and Biological Sciences, University of Karachi, Karachi, 75270, Pakistan*

^e^ *H. E. J. Research Institute of Chemistry, International Center for Chemical and Biological Sciences, University of Karachi, Karachi, 75270, Pakistan*

^f^ *Department of Pharmacognosy and Pharmaceutical Botany, Faculty of Pharmaceutical Sciences, Prince of Songkla University, Hat-Yai 90112, Thailand*

^g^ *Phytomedicine and Pharmaceutical Biotechnology Excellence Center, Faculty of Pharmaceutical Sciences, Prince of Songkla University, Hat-Yai 90112, Thailand*

*Corresponding authors: Pharkphoom Panichayupakaranant, Department of Pharmacognosy and Pharmaceutical Botany, Faculty of Pharmaceutical Sciences, Prince of Songkla University, Hat-Yai 90112, Thailand, Tel. +66-74-288980, Email: [pharkphoom.p@psu.ac.th](mailto:pharkphoom.p@psu.ac.th) and Phisit Khemawoot, Chakri Naruebodindra Medical Institute, Faculty of Medicine Ramathibodi Hospital, Mahidol University, Samut Prakan, 10540, Thailand, E-mail: [phisit.khe@mahidol.ac.th](mailto:phisit.khe@mahidol.ac.th)

^†^These authors contributed equally

**Fig. S1.** Chemical structures of (**A**) lawsone methyl ether and (**B**) lawsone

**Fig. S2.** Superimposition of the co-crystallized and redocked ligand in COX-1 (PDB ID: 3KK6) with an RMSD of 0.6 Å.

**Fig. S3.** TLC of standard LME **(S)** and different fractions **(F_1_, F_2_,** and **F_3_)** obtained during the semisynthesis of LME, visualized under UV light and NaOH or H_2_SO_4_ spray

**Fig. S4.** HPLC chromatograms and absorption spectra of (**A**) standard LME and (**B**) LME obtained *via* semisynthesis.

**Table S1**

Animal disposition and allocation in the acute and subacute toxicity studies

| Study phase | Sex | No. of groups | Animals per group (*n*) | Total animals allocated |
| --- | --- | --- | --- | --- |
| Acute toxicity study | Female | 06 | 03 | 18 |
| Subacute toxicity study | Female | 07 | 05 | 35 |
| Subacute toxicity study | Male | 07 | 05 | 35 |
| **Total** | — | — | — | **88** |

**Table S2**

STopTox predicted 6-pack toxicity endpoints for LME and lawsone

| **Toxicity** | **Prediction** | **Confidence** | **Fragment contribution** |
| --- | --- | --- | --- |
| **LME** | | | |
| Acute inhalation toxicity | Toxic | 53% |  |
| Acute oral toxicity | Non-toxic | 52% |  |
| Acute dermal toxicity | Toxic | 57% |  |
| Eye irritation/corrosion | Non-toxic | 66% |  |
| Skin sensitization | Non-Sensitizer | 60% |  |
| Skin irritation/corrosion | Negative | 70% |  |
| **Lawsone** | | | |
| Acute inhalation toxicity | Non-toxic | 55% |  |
| Acute oral toxicity | Toxic | 68% |  |
| Acute dermal toxicity | Toxic | 66% |  |
| Eye irritation/corrosion | Toxic | 62% |  |
| Skin sensitization | Non-Sensitizer | 60% |  |
| Skin irritation/corrosion | Negative | 80% |  |

**Table S3**

ADMETlab predicted toxicity endpoints for LME and lawsone

| Compound | Property | Confidence | Decision |
| --- | --- | --- | --- |
| **LME** | | | |
|  | Rat oral acute toxicity | 0.498 |  |
|  | Carcinogenicity | 0.824 |  |
|  | Lung toxicity | 0.667 |  |
|  | Hepatoxicity | 0.598 |  |
|  | Genotoxicity | 0.524 |  |
|  | Nephrotoxicity | 0.188 |  |
|  | Neurotoxicity | 0.603 |  |
|  | Ototoxicity | 0.134 |  |
|  | Hematotoxicity | 0.598 |  |
|  | Immunotoxicity | 0.067 |  |
|  | Skin sensitization | 0.917 |  |
|  | Eye corrosion | 0.853 |  |
| **Lawsone** | | | |
|  | Rat oral acute toxicity | 0.61 |  |
|  | Carcinogenicity | 0.729 |  |
|  | Lung toxicity | 0.856 |  |
|  | Hepatoxicity | 0.486 |  |
|  | Genotoxicity | 0.632 |  |
|  | Nephrotoxicity | 0.15 |  |
|  | Neurotoxicity | 0.258 |  |
|  | Ototoxicity | 0.457 |  |
|  | Hematotoxicity | 0.339 |  |
|  | Immunotoxicity | 0.09 |  |
|  | Skin sensitization | 0.952 |  |
|  | Eye corrosion | 0.962 |  |

The green label (	): Excellent, Black label (	): Medium, and the red label (	):  Poor endpoint

**Table S4**

Docking scores of LME, lawsone, and standard inhibitors (aspirin, zileuton, and resveratrol) against cyclooxygenase-I, lipoxygenase, NAD(P)H oxidase, and myeloperoxidase

| PubChem CID | Ligand | Affinity (Kcal/mol) |  | PubChem CID | Ligand | Affinity (Kcal/mol) |
| --- | --- | --- | --- | --- | --- | --- |
| **Cyclooxygenase-I (PDB ID: 3KK6, Resolution: 2.75 Å)** | | |  | **Lipoxygenase (PDB ID: 1N8Q, Resolution: 2.10 Å)** | | |
| 16871 | LME | –5.9 |  | 16871 | LME | –6.6 |
| 6755 | Lawsone | –5.3 |  | 6755 | Lawsone | –5.8 |
| 2244 | Aspirin | –5.6 |  | 60490 | Zileuton | –6.4 |
| **NAD(P)H oxidase (PDB ID: 2CDU, Resolution: 1.80 Å)** | | |  | **Myeloperoxidase (PDB ID: 1DNU, Resolution: 1.85 Å)** | | |
| 16871 | LME | –5.6 |  | 16871 | LME | –4.7 |
| 6755 | Lawsone | –4.7 |  | 6755 | Lawsone | –4.4 |
| 445154 | Resveratrol | –6.1 |  | 445154 | Resveratrol | –5.1 |

PubChem CID: PubChem Compound Identifier and PDB ID: Protein Data Bank Identifier

**Table S5**

RMSD, RMSF, and Rg values of LME complexed with 1DNU, 2CDU, 1N8Q, and 3KK6

| Complex/Apo-protein | Average RMSD | Average RMSF | Average Rg |
| --- | --- | --- | --- |
| LME–1DNU | 0.21 ± 0.01 | 0.12 ± 0.07 | 2.33 ± 0.01 |
| Apo-protein | 0.24 ± 0.03 | 0.12 ± 0.07 | 2.33 ± 0.00 |
| LME–2CDU | 0.17 ± 0.02 | 0.13 ± 0.08 | 2.39 ± 0.01 |
| Apo-protein | 0.18 ± 0.02 | 0.13 ± 0.08 | 2.38 ± 0.01 |
| LME–1N8Q | 0.25 ± 0.02 | 0.14 ± 0.06 | 2.91 ± 0.00 |
| Apo-protein | 0.23 ± 0.02 | 0.13 ± 0.08 | 2.88 ± 0.01 |
| LME–3KK6 | 0.22 ± 0.02 | 0.14 ± 0.07 | 2.41 ± 0.00 |
| Apo-protein | 0.22 ± 0.01 | 0.14 ± 0.07 | 2.43 ± 0.01 |

RMSD: Root mean square deviation, RMSF: Root mean square fluctuation, and Rg: Radius of gyration

**Table S6**

Acute effects of LME and lawsone on the physical and neurobehavioral parameters of female rats observed over 14 days

| Observations | Control | | | |  | LME/lawsone–50 mg/kg | | | |  | LME–300 mg/kg | | | |
| --- | --- | --- | --- | --- | --- | --- | --- | --- | --- | --- | --- | --- | --- | --- |
|  | 1 h | 24 h | 7 D | 14 D |  | 1 h | 24 h | 7 D | 14 D |  | 1 h | 24 h | 7 D | 14 D |
| Physical appearance | N | N | N | N |  | N | N | N | N |  | A | A | A | N |
| Respiratory pattern | N | N | N | N |  | N | N | N | N |  | A | N | N | N |
| Somato-motor activity | N | N | N | N |  | N | N | N | N |  | A | A | N | N |
| Salivation | N | N | N | N |  | N | N | N | N |  | A | N | N | N |
| Fecal consistency | N | N | N | N |  | N | N | N | N |  | A | A | N | N |
| Sleep | N | N | N | N |  | N | N | N | N |  | A | N | N | N |
| Coma | N | N | N | N |  | N | N | N | N |  | A | N | N | N |
| Mortality | 0/3 | 0/3 | 0/3 | 0/3 |  | 0/3 | 0/3 | 0/3 | 0/3 |  | 0/3 | 1/3 | 1/3 | 1/3 |

LME: Lawsone methyl ether, h: Hour(s), D: Day, N: Normal/Not observed, and A: Abnormal/Observed

**Table S7**

Subacute effects of LME and lawsone on the physical and neurobehavioral parameters of male and female rats observed over 28 days

| Observations | Control | | | |  | LME/lawsone–15, 30 or 50 mg/kg | | | |
| --- | --- | --- | --- | --- | --- | --- | --- | --- | --- |
|  | 1^st^ Week | 2^nd^ Week | 3^rd^ Week | 4^th^ Week |  | 1^st^ Week | 2^nd^ Week | 3^rd^ Week | 4^th^ Week |
| Physical appearance | N | N | N | N |  | N | N | N | N |
| Respiratory pattern | N | N | N | N |  | N | N | N | N |
| Somato-motor functions | N | N | N | N |  | N | N | N | N |
| Salivation | N | N | N | N |  | N | N | N | N |
| Fecal consistency | N | N | N | N |  | N | N | N | N |
| Sleep | N | N | N | N |  | N | N | N | N |
| Coma | N | N | N | N |  | N | N | N | N |
| Mortality | 0/5 | 0/5 | 0/5 | 0/5 |  | 0/5 | 0/5 | 0/5 | 0/5 |

LME: Lawsone methyl ether and N: Normal/Not observe

**Table S8**

Different parameters required to calculate acute oral LD_50_ values of LME and lawsone *via* Karber’s method

| Dose | Differential dose (A) | *n** | Mortalities | Mean mortality (B) | A × B |
| --- | --- | --- | --- | --- | --- |
| **LME** | | | | | |
| 50 mg/kg | 0 | 3 | 0 | 0 | 0 |
| 300 mg/kg | 250 | 3 | 1 | 0.5 | 125 |
| 2000 mg/kg | 1700 | 3 | 3 | 2.5 | 4250 |
| **Lawsone** | | | | | |
| 50 mg/kg | 0 | 3 | 0 | 0 | 0 |
| 300 mg/kg | 250 | 3 | 3 | 1.5 | 375 |

*Number of rats per group

**Equation S1**

LD_50_ values for LME and lawsone were calculated *via* following formula:

$$\mathrm{LD}_{50}=\mathrm{LD}_{100}- \frac{\Sigma(A \times B)}{n}$$

Where, “LD_50_” and “LD_100_” are the lethal doses estimated to cause 50% and 100% mortality, respectively, “A” is the difference between two consecutive log-dose increments, “B” is the average mortality rate between two consecutive doses, and “*n*” is the number of animals per test group. The resultant acute oral LD_50_ values for LME and lawsone were calculated to be 542 mg/kg and 175 mg/kg, respectively.

**Fig. S5.** Acute effects of LME and lawsone on (**A**) body weight changes, (**D**) food/water consumption, (**G**) FBG levels, and (**J**) relative organ weight in female rats. Statistical significance was determined by one-way or two-way ANOVA, as appropriate, followed by Tukey’s post-hoc test (**p* < 0.05). Subacute effects of LME and lawsone on (**B** and **C**) body weight changes, (**E** and **F**) food/water consumption, (**H** and **I**) FBG levels, and (**K** and **L**) relative organ weight in male and female rats, respectively. Statistical significance was determined using two-way or three-way ANOVA, as applicable, followed by Tukey's post-hoc test (**p* < 0.05). No statistically significant sex related differences were observed, therefore, the data for male and female rats were presented separately to improve the clarity.

**Fig. S6.** Acute effects of LME and lawsone on the (**A**) liver function, (**D**) kidney function, and (**G**) lipid biomarkers in female rats. Statistical significance was determined using one-way ANOVA followed by Tukey’s post-hoc test (**p* < 0.05). Subacute effects of LME and lawsone on the (**B** and **C**) liver function, (**E** and **F**) kidney function, and (**H** and **I**) lipid biomarkers in male and female rats, respectively. Statistical significance was determined using two-way ANOVA (Treatment × Sex), followed by Tukey's post-hoc test (**p* < 0.05). No statistically significant sex related differences were observed, therefore, the data for male and female rats were presented separately to improve the clarity.

**Fig. S7.** Acute and subacute effects of LME and lawsone on hematological parameters. Acute effects of LME and lawsone on (**A**) blood cell count, (**D**) RBC indices, and (**G**) leucocytes count in female rats. Statistical significance was determined using one-way ANOVA, followed by Tukey’s post-hoc test (**p* < 0.05). Subacute effects of LME and lawsone on (**B** and **C**) blood cell count, (**E** and **F**) RBC indices, and (**H** and **I**) leucocytes count in male and female rats, respectively. Statistical significance was determined using two-way ANOVA (Treatment × Sex), followed by Tukey's post-hoc test (**p* < 0.05). No statistically significant sex related differences were observed, therefore, the data for male and female rats were presented separately to improve the clarity.

**Fig. S8.** Representative photomicrographs of hematoxylin and eosin (H&E)-stained sections of the heart, liver, lungs, kidneys, and spleen from male and female rats in the control and subacute-treatment groups receiving repeated oral doses of LME (50 mg/kg) or lawsone (50 mg/kg) for 28 days. Scale bar = 50 µm.

**Table S9**

Semiquantitative histopathological severity scores of the major organs in rats following acute or subacute oral administration of LME and lawsone

| Compound–Dose | Histopathological severity score | | | | |
| --- | --- | --- | --- | --- | --- |
|  | Heart | Liver | Lungs | Spleen | Kidneys |
| Acute oral toxicity test (OECD-423) | | | | | |
| Control | 0 | 0 | 0 | 0 | 0 |
| LME–50 mg/kg | 0 | 0 | 0 | 0 | 0 |
| LME–300 mg/kg | 0 | 2 | 0 | 0 | 0 |
| LME–2000 mg/kg | 0 | 3 | 0 | 1 | 0 |
| Lawsone–50 mg/kg | 0 | 0 | 0 | 0 | 0 |
| Lawsone–300 mg/kg | 0 | 3 | 0 | 1 | 0 |
| 28-Days repeated oral toxicity test (OECD-407) | | | | | |
| Male | | | | | |
| Control | 0 | 0 | 0 | 0 | 0 |
| LME–50 mg/kg | 0 | 0 | 0 | 0 | 0 |
| Lawsone–50 mg/kg | 0 | 0 | 0 | 0 | 0 |
| Female | | | | | |
| Control | 0 | 0 | 0 | 0 | 0 |
| LME–50 mg/kg | 0 | 0 | 0 | 0 | 0 |
| Lawsone–50 mg/kg | 0 | 0 | 0 | 0 | 0 |

0 = Unchanged, 1 = Mild, 2 = Moderate, 3 = Severe
